# Role of transcriptional regulation in auxin-mediated response to abiotic stresses

**DOI:** 10.3389/fgene.2024.1394091

**Published:** 2024-04-24

**Authors:** Davide Marzi, Patrizia Brunetti, Shashank Sagar Saini, Gitanjali Yadav, Giuseppe Diego Puglia, Raffaele Dello Ioio

**Affiliations:** ^1^ Research Institute on Terrestrial Ecosystems-National Research Council (IRET-CNR), Montelibretti, Italy; ^2^ National Biodiversity Future Center (NBFC), Palermo, Italy; ^3^ Department of Vegetable Crops, Newe Ya’ar Research Center, Agricultural Research Organization, The Volcani Center, Ramat Yishay, Israel; ^4^ Biodiversity Informatics Laboratory, National Institute of Plant Genome Research (NIPGR), New Delhi, India; ^5^ Institute for Agriculture and Forestry Systems in the Mediterranean (ISAFoM), National Research Council of Italy (CNR), Catania, Italy; ^6^ Dipartimento di Biologia e Biotecnologie “Charles Darwin”, Sapienza Università di Roma, Rome, Italy

**Keywords:** global climate change, stress tolerance, transcription factor, auxin, drought stress, salt stress, heavy metals

## Abstract

Global climate change (GCC) is posing a serious threat to organisms, particularly plants, which are sessile. Drought, salinity, and the accumulation of heavy metals alter soil composition and have detrimental effects on crops and wild plants. The hormone auxin plays a pivotal role in the response to stress conditions through the fine regulation of plant growth. Hence, rapid, tight, and coordinated regulation of its concentration is achieved by auxin modulation at multiple levels. Beyond the structural enzymes involved in auxin biosynthesis, transport, and signal transduction, transcription factors (TFs) can finely and rapidly drive auxin response in specific tissues. Auxin Response Factors (ARFs) such as the ARF4, 7, 8, 19 and many other TF families, such as WRKY and MADS, have been identified to play a role in modulating various auxin-mediated responses in recent times. Here, we review the most relevant and recent literature on TFs associated with the regulation of the biosynthetic, transport, and signalling auxin pathways and miRNA-related feedback loops in response to major abiotic stresses. Knowledge of the specific role of TFs may be of utmost importance in counteracting the effects of GCC on future agriculture and may pave the way for increased plant resilience.

## 1 Introduction

Global climate change (GCC) is producing significant environmental disruptions with potentially damaging consequences for the persistence of all life forms across biomes (Bernatchez et al., 2023). The direct consequences of GCC include, among others, extreme temperature waves and water scarcity, which lead to increased soil salinity, and the increase of nonessential elements, among them heavy metals (HMs), thereby increasing the risk of environmental contamination. These abiotic stressors can negatively impact the growth and productivity of plants, functioning below optimal levels (Cramer et al., 2011), which leads to substantial losses in the agricultural economy. Plants cope with these stressful and unfavourable factors by evolving their ability to survive wide-ranging abiotic stresses. To this end, plants have developed elaborate regulatory mechanisms that enable them to withstand adverse growth conditions through biochemical routes that control how plants respond. These pathways include stress detection, signal transmission, and adjustments in physiological parameters to attain varying levels of tolerance (Zhang et al., 2022). Notably, among these factors, several plant hormones facilitate adaptation to abiotic stresses. In this regard, it has been shown that auxin, one of the main phytohormones, regulates plant growth in response to abiotic stresses, such as drought and salinity, influencing root cell activity or differentiation, depending on its different distribution in this organ (Sabatini et al., 1999; Blilou et al., 2005; Dello Ioio et al., 2008; Di Mambro et al., 2017; Kalve et al., 2020; Ghelli et al., 2023). The accumulation of this phytohormone in tissues is mainly modulated by its biosynthetic, signalling, and transport routes. These pathways can be regulated at multiple levels, such as epigenetic, transcriptional, and post-translational (Terrile et al., 2012; Van Der Woude et al., 2019; Marzi et al., 2020; Powers and Strader, 2020). Controlling gene expression is one of the most investigated mechanisms in plants for rapidly and finely adapting transcriptional programmes in response to changes in the environment. Transcription factors (TFs) are proteins that bind to DNA and specifically target cis-acting elements in the promoter region of eukaryotic genes to control their expression (Lelli et al., 2012; Lambert et al., 2018). Previous research on TFs related to auxin homeostasis has highlighted the central role of the antagonistic interaction between auxin response factors (ARFs), most of which are auxin signalling enhancers, and Auxin/INDOLE-3-ACETIC ACID (AUX/IAAs), a large family of auxin co-receptors and transcriptional repressors. Both Aux/IAA and ARFs were shown to play a pivotal role in bridging auxin signalling to that of other phytohormones, such as abscisic acid (ABA). The interaction between auxin and ABA is a key part of fine-tuning of plants responses to abiotic stress. It helps plants deal with drought in a number of ways, such as by closing their stomata to stop water loss (Salehin et al., 2019) or by controlling proline biosynthesis, an amino acid having a highly beneficial role in stress response (Hayat et al., 2012; Zhang et al., 2021). Severe environmental conditions such as drought, salinity, or HM accumulation prompt an oxidative burst through the rise of reactive oxygen species (ROS). These are a group of very reactive molecules that include the superoxide anion (O_2_^·−^), hydrogen peroxide (H_2_O_2_), hydroxyl radical (^·^OH), and singlet oxygen (^1^O_2_). At low levels, these compounds function as signalling components, regulating important metabolic pathways, whereas when their levels increase beyond a ‘safe threshold’, they can be responsible for oxidative damage (Hasanuzzaman et al., 2020; Puglia, 2023). This leads to a number of harmful effects, such as lipid peroxidation, increased membrane permeability, disruption of the antioxidant system, induction of mitochondrial degradation, and DNA and RNA damage (Kurek et al., 2019; Hasanuzzaman et al., 2020). Thus, the regulation of ROS homeostasis is of primary importance in the tolerance to abiotic stresses, and it is achieved by the antioxidative defence systems, including enzymatic and non-enzymatic responses (Hasanuzzaman et al., 2020; Puglia, 2023).

In this study, we present an updated overview aimed at unveiling the importance of auxin regulation in response to abiotic stresses attained through transcriptional regulation of its biosynthetic, signalling, and transport pathways. We will take into consideration novel findings on *Arabidopsis thaliana* model species as well as translational studies on crop species such as tomato and rice. Moreover, in this review, we highlight promising players recently discovered that can provide new tools for the enhancement of plant tolerance to GCC-enhanced abiotic stress effects.

## 2 Auxin biosynthesis, transport, and signalling

**FIGURE 1:**
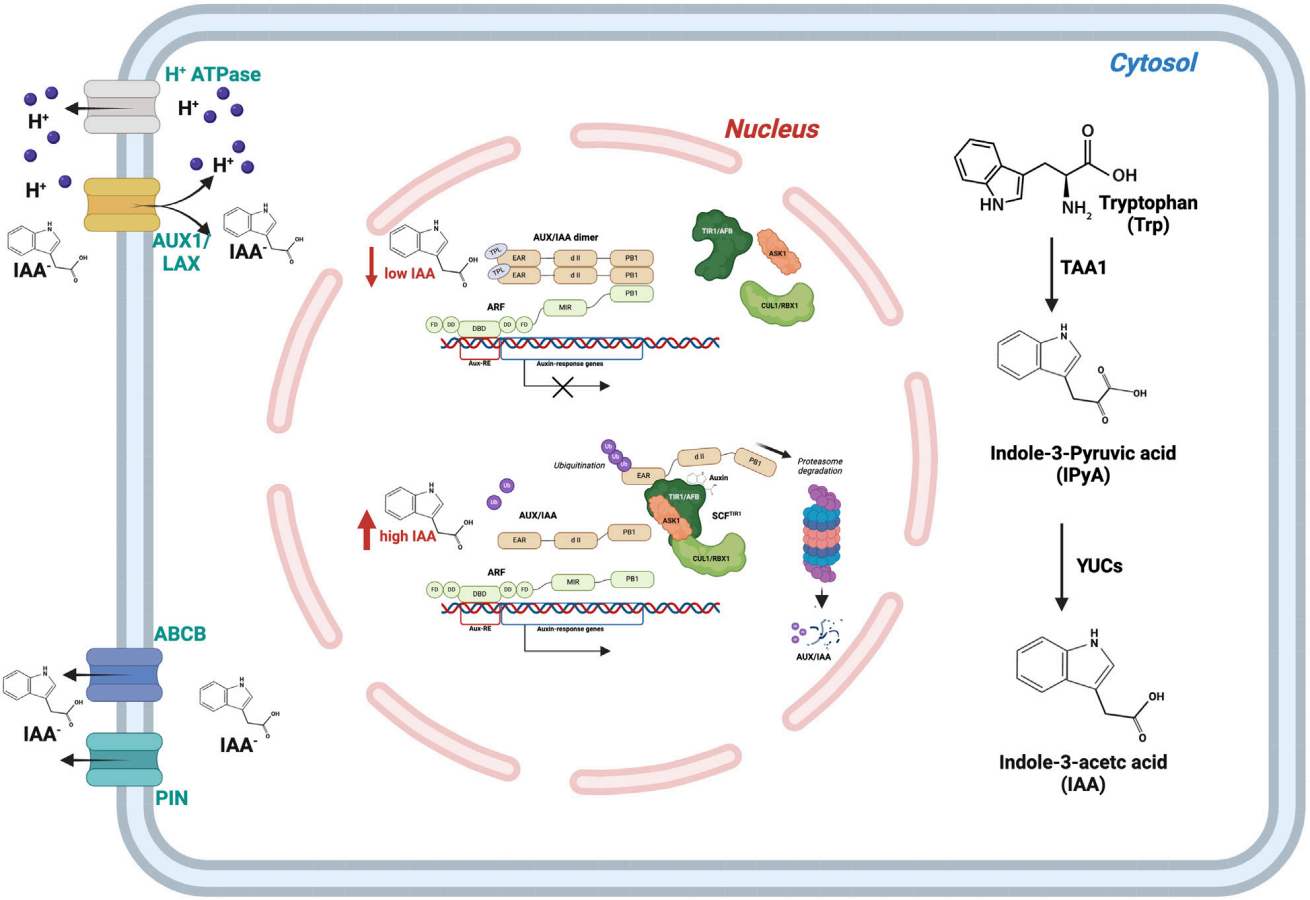
Auxin concentration in the cell is regulated by three different pathways: biosynthesis, transport, and signal transduction. Cytosolic biosynthesis is mainly performed by the TAA1/YUC pathway. Auxin concentration in the cell can be modulated through PIN and ABCB membrane proteins. The auxin signal is transduced through the mediation of ARF-AUX/IAA transcription factors.

The biosynthesis of indole-3-acetic acid (IAA), the main natural auxin, occurs via two pathways, one dependent on tryptophan (Trp) and another Trp independent (Cao et al., 2019; Sato et al., 2022). Tryptophan is produced from chorismate through the intermediate indole-3-glycerol phosphate (IGP), with the anthranilate synthase alpha subunit (ASA) serving as the initial enzyme in the tryptophan synthesis process. Downstream of Trp production, in the indole-3-acetaldoxime (IAOx) pathway, indole-3-acetonitrile (IAN) is converted to IAA via nitrilase (NIT) (Zhao, 2010). While in the TRYPTOPHAN AMINOTRANSFERASE OF ARABIDOPSIS/YUCCA (TAA/YUC) pathway, the structural enzyme tryptophan aminotransferase (TAA1), through an amino-transfer reaction, converts Trp to indole-3-pyruvic acid (IPyA), which is then decarboxylated to form IAA by flavin-containing monooxygenases (FMOs) of the YUCCA (YUC) type (Figure 1). Recent studies have shown that the reversible TAA1 reaction, which prevents over- or under-accumulation of IPyA based on different Km values of Trp and IPyA, finely regulates this two-step biosynthetic pathway (Sato et al., 2022). This mechanism allows the concentration of IPyA to be kept low so that it can only be converted enzymatically to IAA by the irreversible and rate-limiting reaction catalysed by YUC, avoiding the non-enzymatic generation of IAA (Cao et al., 2019; Sato et al., 2022).

The uneven distribution of auxin level is attained by the directional auxin transport mediated by specific families of influx, AUXIN RESISTANT1/LIKE AUX1 (Aux/LAX) (Swarup and Bhosale, 2019), or efflux channel proteins, PIN-FORMED (PIN) (Křeček et al., 2009; Nodzyński et al., 2016) (Figure 1). The precise positioning of these auxin channels orients auxin flux and creates heterogeneity for IAA distribution. PIN protein activity can be regulated at many levels, including regulation of transcription, protein degradation, subcellular trafficking (endocytic recycling and polarised targeting), and transport activity (Křeček et al., 2009; Kumar et al., 2024).

Auxin, as a signalling molecule, plays an instrumental role in plant growth and developmental processes at varying concentrations. The most known molecular mechanism for auxin-mediated gene expression is based on the TRANSPORT INHIBITOR RESPONSE1/AUXIN SIGNALLING F-BOX PROTEINS (TIR1/AFBs) pathway. The TIR1/AFB pathway primarily involves three components: the (SKP1-CUL1-F-box) SCFTIR1/AFB receptor complex, the Aux/IAA repressor proteins, and ARFs. The latter can be either transcriptional activators (class A ARFs) or transcriptional repressors (class B/C ARFs). In the absence of auxin, the activity of the transcription factor ARF is toned down by the transcriptional repressor Aux/IAA protein (Leyser, 2018). In the presence of auxin, TIR1/AFB proteins bind to the transcriptional repressors Aux/IAA inducing their degradation via the 26S proteasome, allowing transcriptional regulation by ARFs (Figure 1).

Auxin can also be perceived in cells by the apoplastic receptor ABP1 (AUXIN BINDING PROTEIN1) and its homologues ABL1 and 2 (ABP1 LIKE1 and 2). Those proteins bind auxin together with the Transmembrane Receptor Kinases (TMKs), inducing an ultrafast phospho-response (Friml et al., 2022; Yu et al., 2023). TMK proteins phosphorylate also plasma membrane H^+^-ATPases, important regulators of growth (Pacifici et al., 2018; Lin et al., 2021), and ABA INSENSITIVE 1 and 2 (ABI1 and ABI2), negative regulators of Abscisic Acid response (Yang et al., 2021). Although this signalling pathway is newly discovered and has not yet been correlated with abiotic stress responses, these data permit speculation that the ABP1/TMK pathway might be primarily involved in the modulation of growth in response to abiotic stresses.

## 3 GCC is leading to harsh environmental conditions that induce stress responses in plants

Salinity and drought are among the main abiotic stressors that impact plant health worldwide. Both affect soil water potential, leading to insufficient water uptake by plant roots and causing ion imbalance (Cruz de Carvalho, 2008; Mickelbart et al., 2015; Ribba et al., 2020; Verma et al., 2022). Besides, drought leads to a progressive increase of HMs in soil, affecting plant growth (Tipping et al., 2003; Lequeux et al., 2010; Stirling et al., 2020). In this framework, the auxin response serves as a crucial hormone for the adaptation of plants to salinity, drought, and HM stress (Shi et al., 2014; Ribba et al., 2020), linking ROS signalling to physiological development. Indeed, these abiotic stresses trigger a significant increase in ROS, causing changes in many crucial cellular metabolic processes and reactions (Ahanger et al., 2017). Strategies to cope with these changes include maintaining ROS equilibrium, controlling stomata closure, generating proline, and adjusting root growth and structure (Dat et al., 2000; Geng et al., 2013; Verma et al., 2022; Scintu et al., 2023).

### 3.1 Modulation of auxin biosynthesis promotes tolerance to drought and salinity stress

**FIGURE 2:**
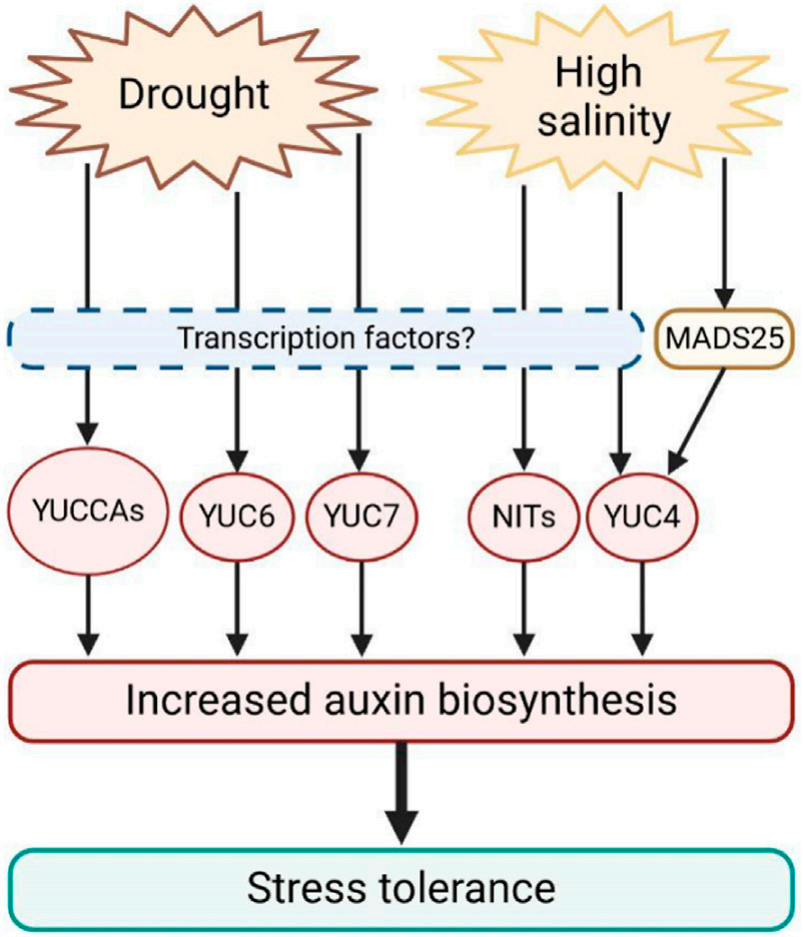
Schematic representation of the expression of auxin biosynthetic genes induced by drought and salt stress in plants. Drought and salt promote the transcription of YUCCAs and NITs genes, which in turn induce auxin biosynthesis. Increased auxin content stimulates stress tolerance. However, transcription factors translating stress signals to auxin biosynthesis still remain largely unknown.

Plants cultivated in salty environments exhibited elevated levels of YUC4, NIT1 and NIT2, which promote auxin production. Arabidopsis *NIT2* overexpressing lines have higher auxin levels only when grown under saline conditions, suggesting a specific role for this enzyme (Cackett et al., 2022). At the same time, auxin biosynthesis also modulates drought responses. Indeed, activation-tagged Arabidopsis mutants *yuc6-1D* and *yuc7-1D* are resistant to drought stress, while *YUCCA6* and *YUCCA7* loss-of-function mutants are more sensitive to water deficiency (Lee et al., 2012; Kim et al., 2013). Transgenic plants of potato (*Solanum tuberosum* cv. *Jowon*) and poplar (*Populus alba × P. glandulosa*) overexpressing the Arabidopsis gene *YUCCA6* showed enhanced drought tolerance, reduced water loss, and decreased levels of ROS (Kim et al., 2013; Ke et al., 2015). Furthermore, Arabidopsis *yuc1 yuc2 yuc6* triple mutants showed decreased auxin production, reduced drought stress resistance, and increased ROS levels (Shi et al., 2014), pointing out that an increase in auxin biosynthesis stimulates stress tolerance. However, a detailed characterization of transcription factors regulating *YUCCAs* expression in response to abiotic stress still lacks. Only recently, it was suggested that in rice (*Oryza sativa*)*,* the MADS-box transcription factor OsMADS25, whose overexpression confers salinity tolerance, induces the expression of *OsYUC4,* promoting auxin biosynthesis to achieve stress resistance (Xu et al., 2018). This evidence further underlines the central role of auxin biosynthesis in salt and drought stress perception and tolerance (Figure 2).

### 3.2 Tolerance to drought and salinity stress requires repression of auxin signalling

Recently, it was demonstrated that in Arabidopsis, the auxin repressors IAA5, IAA6, and IAA19 positively regulate tolerance to drought stress, promoting stomata closure through the repression of the ABA-responsive transcription factor ABA OVERLY SENSITIVE3 (*ABO3*) expression. On the other hand, *iaa5 iaa6 iaa19* triple mutants exhibit reduced drought tolerance, fail to close stomata, and show increased *ABO3* transcript levels in drought conditions (Salehin et al., 2019). In rice, *OsIAA20* RNAi transgenic lines showed decreased proline and chlorophyll content, a significant reduction in stomata closure, and downregulation of the ABA-responsive gene *OsRab21*, which is involved in drought stress tolerance. Accordingly, transgenic plants overexpressing *OsIAA20* exhibited improved tolerance to abiotic stress and increased expression of *OsRab21* (Zhang et al., 2021). Overexpression of *OsIAA18* triggers enhanced salt and drought tolerance, increases ABA biosynthesis and signalling, and promotes proline biosynthesis (Wang F. et al., 2021). Drought and ABA treatments induce the transcription of *OsIAA6*, while *OsIAA6* overexpressing lines show improved drought tolerance (Jung et al., 2015). All these findings support the idea that *Aux/IAA* genes function as hubs integrating physiological development and molecular responses to environmental changes, highlighting that the upregulation of *Aux/IAA* mainly enhances plants tolerance to drought stress.

In parallel, upon drought and salt perception, the expression of *ARFs* may vary in positive or negative ways (Bouzroud et al., 2018). Generally, it is thought that their downregulation improves plants resistance to stress. Silencing of *ARF2* in tomato (*Solanum lycopersicum*) triggers the upregulation of ROS scavenger and proline biosynthesis genes, conferring enhanced tolerance to abiotic stress (El Mamoun et al., 2023). Consistently, Arabidopsis *arf2* knockdown mutants accumulate ABA, resulting in increased stomata closing, reduced leaf transpiration, and improved stress tolerance (Meng et al., 2015). In addition, in tomato, *arf4* loss of function induces the expression of the ABA-related genes *SlABI5* and *SlSCL3* and shows significant alterations in stomata morphology and closing (Chen et al., 2021). Antisense and CRISPR/Cas9 downregulation of *SlARF4* promote root development, higher tolerance to salt and osmotic stress, reduced stomatal conductance, and increased leaf relative water content, corroborating the notion that *SlARF4* negatively regulates tolerance to salt and osmotic stresses (Bouzroud et al., 2020). Moreover, the loss of *AtARF7* and *AtARF19*, two well-known modulators of lateral root development, triggers a significantly reduced sensitivity to osmotic stress (Kalve et al., 2020).

**FIGURE 3:**
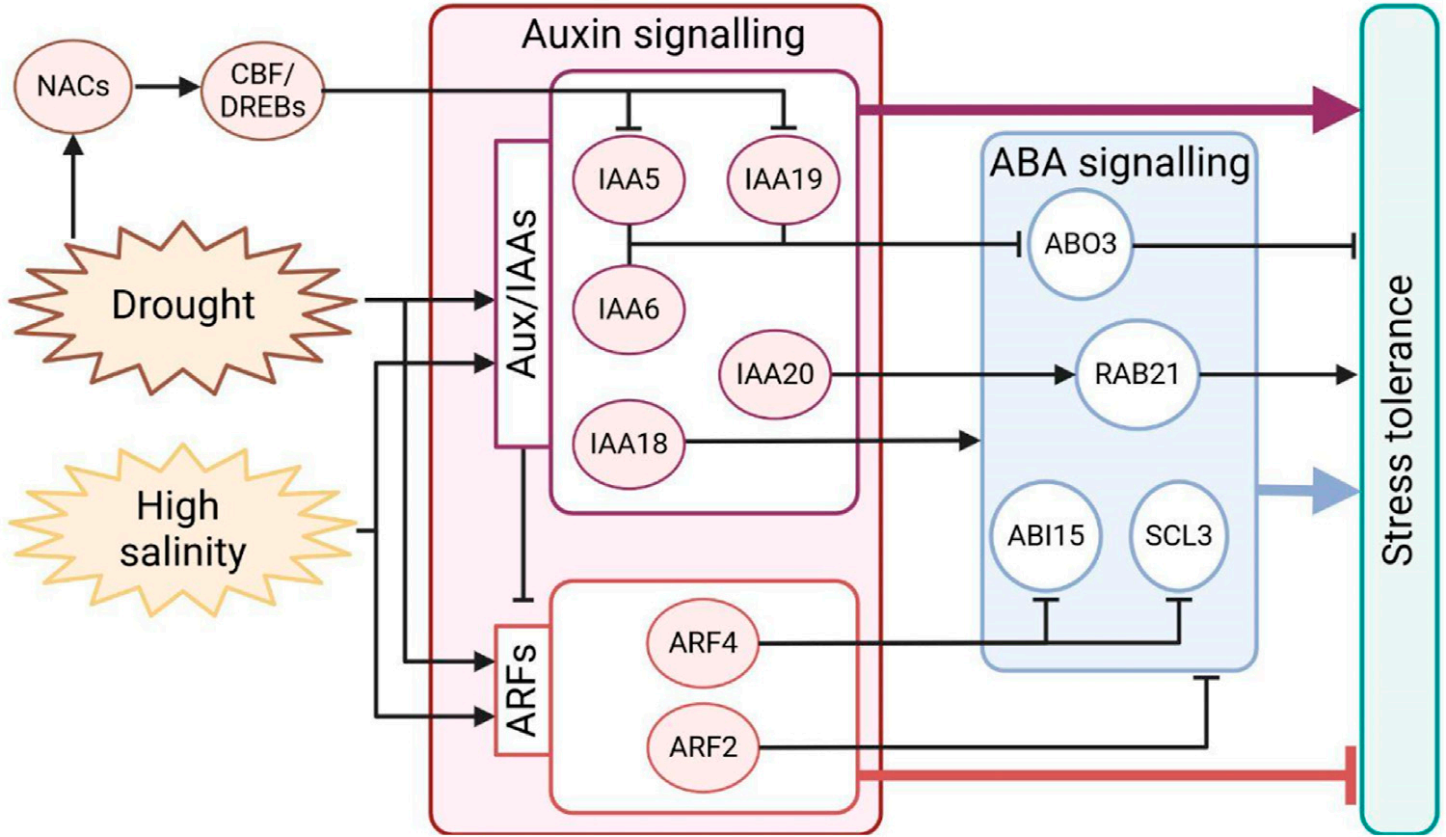
Schematic representation of auxin signalling regulation during drought and salt stress in plants. Both stresses promote the expression of the auxin-responsive genes *Aux/IAAs* and *ARFs*; however, the effective repression of ARFs could promote stress tolerance. NACs and DREBs are activated by stress, interact with each other, and modulate auxin signalling to achieve stress tolerance. ABA and auxin are connected by a tight interplay, partially illustrated in the figure. Expression of genes responsive to ABA can be induced or repressed by the auxin signalling component during stress responses. Arrows indicate positive regulation, and T-bars indicate negative regulation.

Among transcription factors, C-repeat/dehydration-responsive element binding proteins (CBF/DREB) play an important and positive role in stress tolerance. In particular, *CBF/DREB* are upregulated in response to abiotic stress, and their overexpression is associated with drought tolerance (Lata and Prasad, 2011; Dong et al., 2017). Several CBF/DREBs directly promote the transcription of *Aux/IAAs*, such as *IAA5* and *IAA19*, in response to desiccation stress in Arabidopsis (Shani et al., 2017). These findings further support the notion of Aux/IAAs functioning as an intersection for the integration of stress responses (Figure 3).

Furthermore, the expression of transcription factors from the *NAC* family–*NAM* (no apical meristem) and *ATAF1/2* and *CUC2* (cup-shaped cotyledon)–is induced by abiotic stress, and they modulate gene transcription under drought conditions (Shao et al., 2015). It was shown that soybean (*Glycine max*) *GmNAC20* and *GmNAC11* overexpression in Arabidopsis plants enhances salt tolerance, promotes *DREB1A* expression, and modulates auxin signalling by acting on *ARF7* and *ARF19* expression (Hao et al., 2011). Similarly, it was demonstrated that overexpression of pepper (*Capsicum annuum*) *NAC46* in Arabidopsis transgenic lines enhances resistance to drought and salt, increasing the expression of *IAA4* (Ma et al., 2021). It is worth mentioning that a tight connection exists between auxin, *NAC* transcription factors, and abiotic stress responses, as *NAC2* is induced by salt stress and its expression is abolished in *tir1-1* mutants in Arabidopsis (He et al., 2005).

### 3.3 Auxin transport as a key regulator for salt stress tolerance

Auxin activity and transport have been widely associated with root development. In this organ, auxin distribution is strictly related to cell developmental stages, as a maximum of auxin maintains stem cell activity, whereas a minimum prompts cells to differentiate (Sabatini et al., 1999; Blilou et al., 2005; Di Mambro et al., 2017). Being in direct contact with soil, roots are the first organ in plants to perceive drought and salinity stresses and to be subjected to stress-dependent growth inhibition. Auxin regulates root growth, promoting cell division in the root meristem, a region located at the root tip where a set of self-renewal stem cells support continuous growth (Di Mambro et al., 2018). It has been recently shown that salinity stress represses root development, promoting the exit of cells from the meristem and, hence, cell elongation and differentiation, activities dependent on variation in polar auxin transport (Scintu et al., 2023). For the reasons above, roots have been largely utilised as model systems to study the effect of drought and salt stresses on auxin transport. First evidence that auxin distribution varies in response to salinity stress has been given by the indirect auxin sensor DR5, which shows a maximum of activity in the root stem cell niche. DR5 activity is largely decreased by salt exposure, most likely due to a decreased expression of the polar auxin transporter PIN1, 2, and 3 (Liu et al., 2015; Fu et al., 2019; Smolko et al., 2021). Little is known about the molecular basis governing the salt-dependent inhibition of *PINs* expression. Recently, it has been shown that short exposures to salt stress promote the expression of the SHORT HYPOCOTYL2 *(SHY2)/IAA3* gene, a negative regulator of the polar auxin transport in the root meristem, via induction of cytokinin biosynthesis in the meristem (Dello Ioio et al., 2008; Dello Ioio et al., 2012; Scintu et al., 2023). This finding provides an enthralling possibility that, at first, salt represses plant growth by inhibiting auxin signalling and, hence, auxin distribution, altering cell developmental stages. In the future, it will be key to understand whether and how variation in auxin distribution in roots might infer shoot organ growth inhibition in response to salt stress.

**FIGURE 4:**
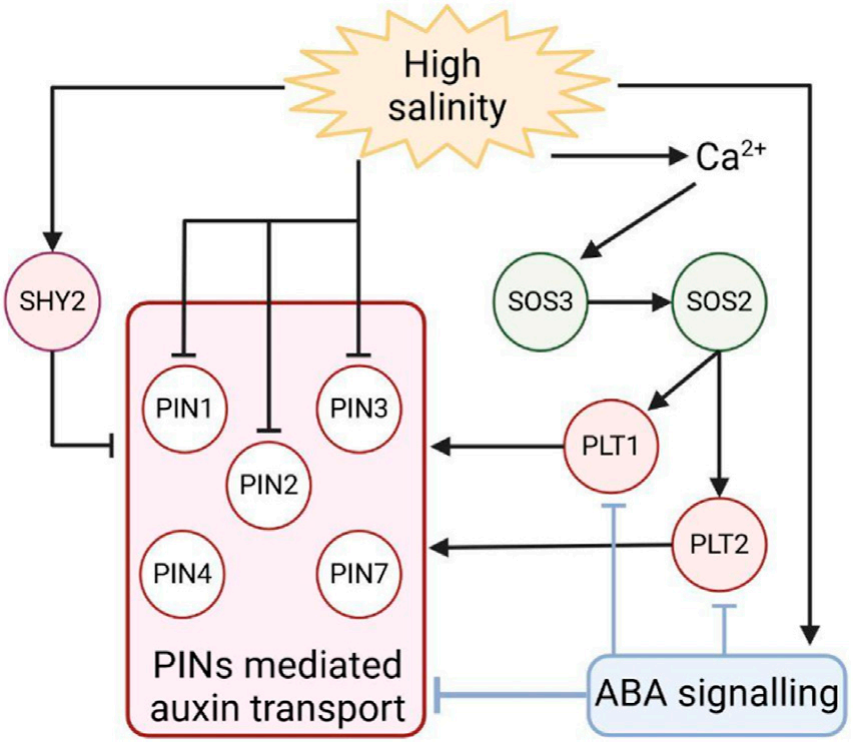
Schematic representation of PINs-mediated auxin transport regulation during salt stress. Responses to salt treatment involve different pathways that regulate the expression of PINs to modulate auxin distribution in roots. Arrows indicate positive regulation, and T-bars indicate negative regulation.

Once perceived salt stress, root development adapts to this re-establishing growth. The Salt Overly Sensitive (SOS) pathway is pivotal in this process, as *sos* mutants show hypersensitivity to salt stress (Yang and Guo, 2018). Over salt exposure, plant cells increase cytosolic Ca^2+^ concentration, leading to the activation of the calcium-binding protein SALT OVERLY SENSITIVE 3 (SOS3) (Yang and Guo, 2018). SOS3 recruits on the plasma membrane the serine/threonine protein kinase SOS2 that activates the Na^+^/H^+^ antiporter SOS1 via phosphorylation, decreasing the intracellular Na^+^ concentration (Yang and Guo, 2018). Interestingly, *sos* mutants oversensitivity to salt stress is partly dependent on PIN regulation by this stress (Zhao, 2018). It has been recently found that SOS2 phosphorylates and stabilises in response to the salts PLT1 and PLT2 (Hao et al., 2023), two auxin-dependent transcription factors regulating auxin distribution via the promotion of *PINs* expression (Blilou et al., 2005). These data open the possibility that plants adapt to salt stress by regulating PIN-dependent auxin distribution, hence promoting growth in adverse conditions (Figure 4). Whether this mechanism is favourable only for root development is still a matter of discussion.

The maintenance of root growth under water stress conditions has been largely studied in rice and tomato. This is mediated by a synergistic action of ABA and auxin, in which the former hormone accumulates first in the root tip and modulates auxin transport through regulation of AUX/LAX and PIN, which enhances proton secretion to preserve the root growth from severe water stress. In this context, the involvement of transcription factors in auxin transport remains to be fully unveiled, but some evidence gives them a role in fine-tuning the response to drought and salinity stress. The WRKYs transcription factors are reported as important regulators of abiotic stress tolerance (Jiang and Deyholos, 2006; Zhou et al., 2008; Niu et al., 2012; Song et al., 2016). In tomato, overexpression of *SlWRKY3* provides tolerance to salinity and, consequently, reduced oxidative stress and proline contents compared to wild-type. Interestingly, those plants show upregulation of LAX3 (auxin influx carrier) proteins, providing a role for this auxin influx carrier in salt and drought stress tolerance (Hichri et al., 2017).

Salt and drought stress inhibit root complexity, repressing lateral root elongation via ABA (De Smet et al., 2003; Lu et al., 2019). It has been shown that the emergence and elongation of lateral roots are strictly dependent on the formation of an auxin maximum that is guided by PINs cellular localization (Cavallari et al., 2021). Long exposure to high salinity stress promotes ABA synthesis and signalling in roots (Geng et al., 2013). Interestingly, ABA is able to repress the expression of several PIN proteins, such as *PIN1, 3, 4*, and *7*, and the expression of *PLT1* and *PLT2* in both the elongation zone of the primary root and lateral roots (Promchuea et al., 2017). As lateral root formation also depends on auxin accumulation and distribution in the meristem, the ABA/auxin module might represent a checkpoint for defining root architecture in response to salt stress. Further studies will elucidate the intricate connection between these two hormones to regulate root architecture in response to salinity and drought stress.

### 3.4 miRNAs represent a central hub in auxin-dependent regulation of drought and salt stress responses

In recent years, ∼21–24 nucleotide-long single-strand microRNAs (miRNAs) have emerged as fundamental players in plant development control (Bertolotti et al., 2021). Recent studies on Arabidopsis have shown that the activity of these small macromolecules is required for regulating the response to several environmental stresses, including salt, drought, and HMs stresses (Zhang et al., 2022). Interestingly, numerous of these stress-related miRNAs target the mRNA of auxin-related genes. The first evidence of auxin-related miRNAs regulating development comes from miR393, targeting *TIR1/AFB* auxin receptors (Chen et al., 2011). The expression of this miRNA is induced in response to several stresses, including salt and drought stress, in several crops, suggesting a fine-tuning mechanism to control growth in response to these stresses (Chen et al., 2015; Bai et al., 2017; Yuan et al., 2019). miR160 targeting *ARF10, 16*, and *17* has been found to be a central hub for mediating plastic development after exposure to several stresses in a multitude of species, such as wheat, rice, and barley (Reinhart et al., 2002; Liu et al., 2010; Hao et al., 2022).

In most of the species, drought stress promotes *MIR160* expression, dampening *ARFs* expression levels; on the contrary, salt stress represses its expression (Hao et al., 2022). When exposed to high salinity, *MIR160* transcription is inhibited in rice and in citrus plants (Huang et al., 2016; Dubey et al., 2020; Zhou et al., 2020). The regulation of *MIR160* in response to stresses highlights the importance of auxin activity in plant adaptation to the environment, as this is fundamental for both regulating lateral root development and counteracting ROS accumulation (Bertolotti et al., 2021). Interestingly, auxin-related miR167 is involved in *ARF6* and *ARF8* post-transcriptional regulation, and miR390 is involved in the generation of tasiRNA (TAS3-derived trans-acting short-interfering RNA), which targets *ARF2, ARF3*, and *ARF4* (Montgomery et al., 2008; Marin et al., 2010) and has been shown to be regulated by abiotic stresses (Wang et al., 2005; Gutierrez et al., 2009; Khan et al., 2011).

In Arabidopsis, *MIR167* expression is upregulated in response to most of the stresses, but in monocots such as maize, rice, and wheat, it is downregulated (Zhang N. et al., 2018), pointing out how the different ARFs acquired specific roles during evolution.

Several miRNAs whose expression responds to stresses are indirectly affecting auxin dynamics. An example of this is given by miR165 and 166, targeting the transcription factors *HOMEODOMAIN LEUCINE ZIPPER III (HD-ZIPIII)* (Bertolotti et al., 2021). HD-ZIPIIIs regulate both the expression of *ARF5* and the synthesis of cytokinin (Dello Ioio et al., 2012; Müller et al., 2016), which downregulates the expression of PIN genes in the root. *MIR165* and *166* expression is tightly linked to ABA regulation (Yan et al., 2016; Ramachandran et al., 2018; Yang et al., 2019), constituting a link between auxin, cytokinin, and this hormone. Interestingly, over-expression and downregulation of these miRNAs via MIMICRY technology confer tolerance to salt and/or drought stresses (Jia et al., 2015; Scintu et al., 2023).

The exact role of these microRNAs in response to stresses is still a matter of discussion; however, the reported examples highlight the intrinsic nature of these macromolecules in the regulation of mRNA target dosages (Scintu et al., 2023), a field of research that might represent a crucial point for the generation of plants tolerant to abiotic stresses.

### 3.5 Auxin contributes to heavy metals tolerance

Water scarcity is among the most critical effects induced by GCC. Its persistence can lead to an increase in ion concentrations, determining a rise in HMs in soil. Indeed, drought increases the amount of nonessential elements such as aluminium (Al), arsenic (As), cadmium (Cd), and selenium (Se), promoting their accumulation in soil and causing detrimental consequences for plants. Negative effects include low biomass production, chlorosis, reduced photosynthesis, altered water balance, and nutrient assimilation (Finnegan and Chen, 2012; Sun et al., 2014; Ali and Gill, 2022). On the other hand, metals such as zinc (Zn), manganese (Mn), and nickel (Ni) have been classified as essential micronutrients that play a positive role in plant growth when present at optimal concentrations. However, excessive uptake could affect metabolic processes, leading to adverse effects on plants (Hassan et al., 2019; Li et al., 2019; Kaur and Garg, 2021).

**FIGURE 5:**
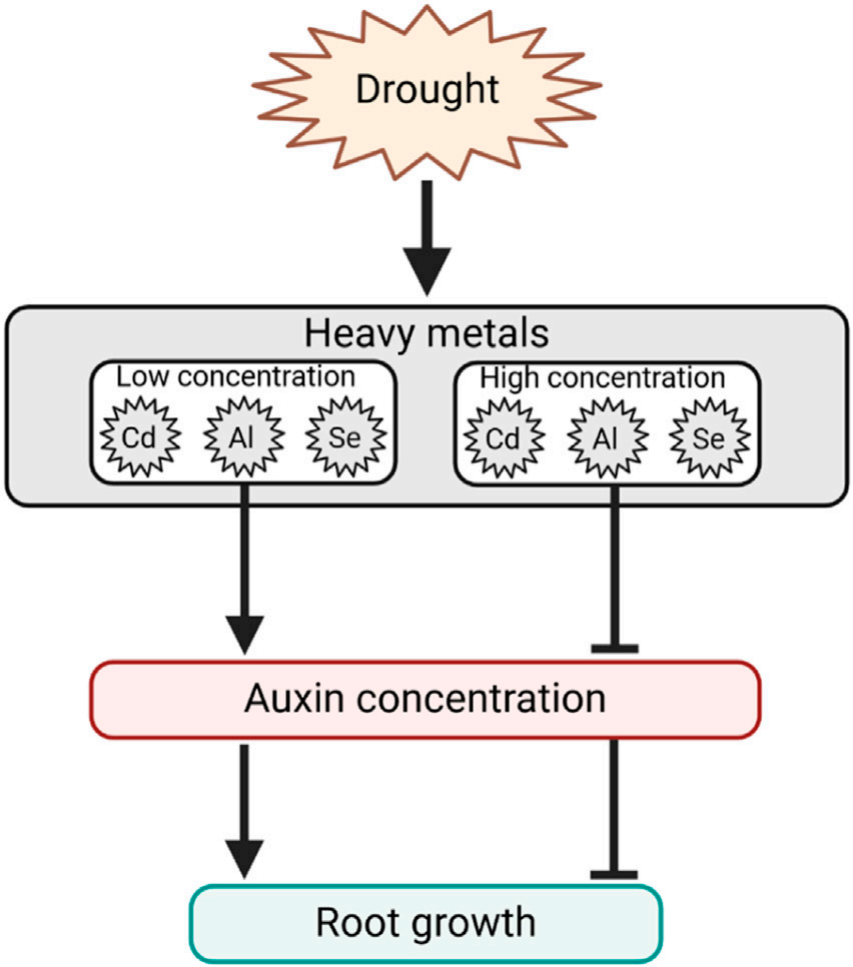
Hypothesized HMs dose-dependent response model regulating auxin concentration and, consequently, root growth. Arrows indicate positive regulation, and T-bars indicate negative regulation.

The primary morphological response related to HMs stress is associated with reduced primary root length, decreased lateral root proliferation, and reduced leaf area. These morphological responses are associated with a deregulation of auxin homeostasis and gene expression in Arabidopsis, in which auxin is a crucial regulator of primary root growth and lateral root initiation and elongation (Overvoorde et al., 2010). However, the effect of HMs on root growth varies depending on the element and its concentration. For example, it was found that in the presence of mild Cd, auxin accumulation occurs in the root tips of Arabidopsis and barley, while at high Cd, auxin levels decrease (Hu et al., 2013; Demecsová et al., 2020). Similarly, the presence of Al promotes auxin accumulation in rice root apex, determining the inhibition of its elongation (Wang et al., 2019). Low and high concentration of Se, respectively, increased or decreased the auxin content in both the root and shoot of tobacco seedlings (Luo et al., 2019). This is due to both the increased expression of genes involved in the IAA biosynthetic pathway, as well as the enhanced activity of IAA oxidase leading to auxin catabolism, alongside the involvement of AUX transporters, which contribute to root auxin depletion. Altogether, these findings suggest that the auxin distribution can highly vary in relation to plant species, chemical elements, and concentrations (Figure 5). Interestingly, a mild or high accumulation of the element usually leads to a rise or inhibition of auxin content and, consequently, plant growth. This response at low doses of toxic substances, also described as hormesis, results in a stimulatory effect on plant growth and can be used to further investigate the underlying molecular mechanisms during stress responses (Calabrese et al., 2016; Małkowski et al., 2020).

#### 3.5.1 HMs differentially regulate auxin biosynthesis

**FIGURE 6:**
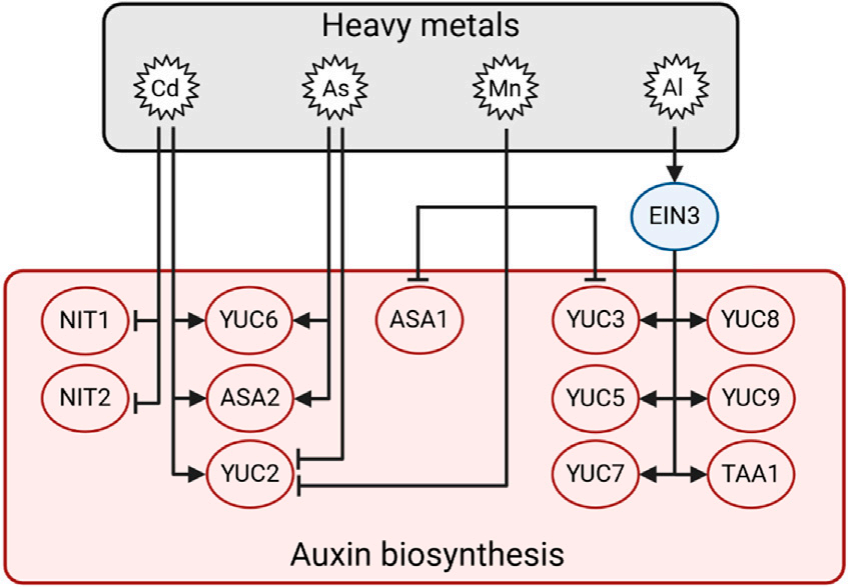
Illustration of the proposed model underlying the auxin biosynthetic pathway in the presence of Al, As, Cd, and Mn. Arrows indicate positive regulation, and T-bars indicate negative regulation.

Recently, it was shown that different YUCCAs are involved in responses to HMs, which can even antagonise each other’s effects depending on the plant species (Yan et al., 2016). In Arabidopsis hypocotyls, Cd stimulates the expression of *AtYUC6* while As suppresses it, leading to the promotion and inhibition of auxin production, respectively (Fattorini et al., 2017). Conversely, *OsASA2* and *OsYUCCA2* transcripts are upregulated by Cd and As in rice roots, which grow less than control ones (Ronzan et al., 2018). In addition, Arabidopsis *yuc6-1D* mutants are more tolerant to Ni stress than control plants (Cha et al., 2022). In tobacco (*Nicotiana tabacum*), Cd stress altered the auxin gradient by affecting auxin biosynthesis genes *NtYUCCA6*, *8*, and *9* (Luo et al., 2019). The ethylene-responsive transcription factor ethylene-insensitive 3 (EIN3) induces the expression of *AtYUC3, 5, 7, 8, 9* and *AtTAA1* upon Al treatment, triggering the repression of root development in Arabidopsis (Liu et al., 2016; Yang et al., 2019). On the other hand, *AtYUC2, 3* and *AtASA1* expression is downregulated by manganese (Mn), which represses root elongation (Zhao et al., 2017). In sorghum (*Sorghum bicolor*), quick responses to Cd include increased expression of auxin biosynthetic genes such as *SbNIT1* and *SbNIT2*, while their expression is significantly repressed after 5 days of Cd treatment (Zhan et al., 2017). Overall, these data indicate that auxin biosynthesis is differentially regulated by different HMs and that it could be induced or repressed depending on the element (Figure 6).

#### 3.5.2 HMs affect auxin transport, impairing auxin homeostasis

**FIGURE 7:**
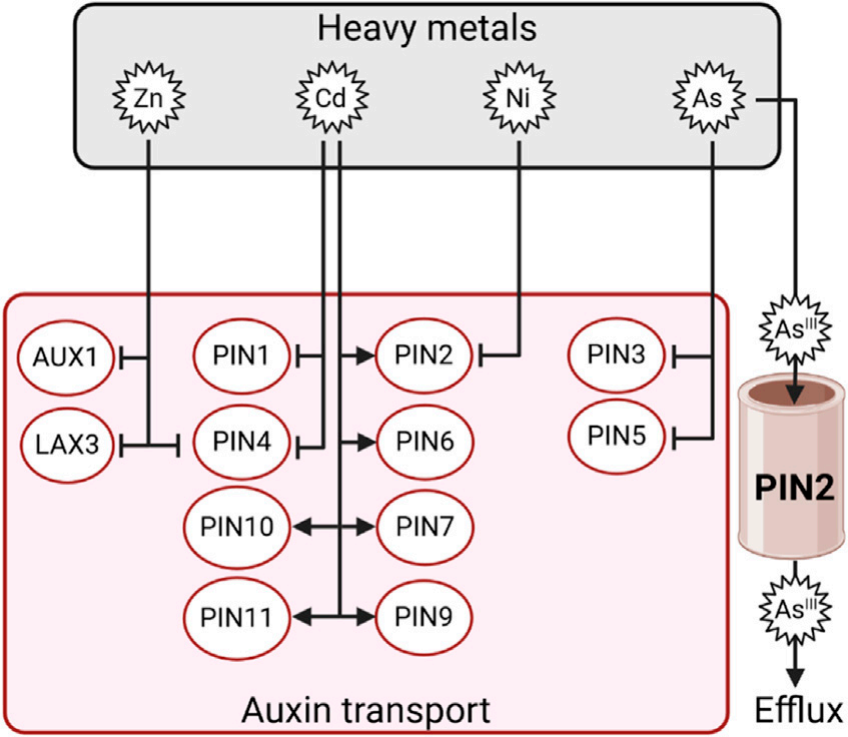
Schematic representation of the differential regulation of auxin transporters by Zn, Cd, Ni, or As. Arrows indicate positive regulation, and T-bars indicate negative regulation.

Besides biosynthesis, HMs also impair auxin transport, affecting its spatial distribution and homeostasis. Indeed, Cd induces a downregulation of *PIN1* and *PIN4* expression in tobacco (Luo et al., 2019). Arsenic caused downregulation of *PIN3* and *PIN5* expression in both the roots and shoots of Brassica plants (Praveen et al., 2019). Similarly, Ni downregulated the expression of *PIN2* and inhibited the shoot-directed distribution of auxin in Arabidopsis (Lešková et al., 2020). *PIN2* overexpression in rice results in the restoration of basipetal IAA transport and inhibits ROS generation in Al-stressed plants (Wu et al., 2014). An interesting finding on PIN2 comes out from studies in Arabidopsis. In this species, *pin2* loss-of-function mutants show hypersensitivity to As(III). Surprisingly, *pin2* plants accumulate more arsenic species in root apices compared with wild-type seedlings, suggesting that PIN2 may function as a specific As(III) efflux transporter and not only as an auxin efflux facilitator (Ashraf et al., 2020). Root growth is altered in Arabidopsis exposed to Zn, which affects the expression of *AUX1, LAX3* (Sofo et al., 2017), and *PIN4* (Zhang P. et al., 2018), resulting in impaired auxin homeostasis (Wang J. et al., 2021). Altogether, these findings give an overview of the complex interaction existing between HMs and auxin in plants, which becomes even more intricate as other actors, such as ROS, play prominent roles in this crosstalk (Figure 7).

#### 3.5.3 Auxin signalling during HMs stress is mediated by auxin-ROS crosstalk and miRNAs

The toxic effects of HMs ions are characterised by a tight auxin-ROS interplay in Arabidopsis, which is essential for stress response (Wang et al., 2015). Their phytotoxicity depends mainly on their high redox activity, which impairs the balance between the generation and degradation of ROS, leading to oxidative stress (Shahid et al., 2014).

Rice mutants for the auxin influx transporter *aux1* appear more sensitive to Cd stress due to the higher accumulation of ROS (Yu et al., 2015). In the roots of sorghum, Cd stress affects auxin and ROS homeostasis, triggering a decrease in root length proportional to the increase in Cd content in the medium. (Zhan et al., 2017). Among auxin transporters, Arabidopsis *aux1*, *pin1,* and *pin2* mutants were significantly sensitive to As (III) and developed shorter roots. On the other hand, *AUX1* was shown to be involved in the production of H_2_O_2_ (Krishnamurthy and Rathinasabapathi, 2013), which is known to be a molecular link between oxidative stress and auxin. H_2_O_2_ activates ANP1 (NPK1-like protein kinase), a specific class of stress-induced mitogen-activated protein kinases (MAPKs) upregulated by oxidative stress and able to repress auxin responsive promoter activity, causing displacement in auxin homeostasis (Kovtun et al., 2000). Interestingly, transcriptomic analysis on Kentucky bluegrass (*Poa pratensis*) treated with Cd revealed the contemporary deregulation of MAPKs signalling pathway and auxin-responsive genes such as *Aux/IAA* and *ARFs* upon stress perception (Xian et al., 2020). In parallel, the interactions between hub TFs and differentially expressed genes were investigated, and MADS25 was found to potentially interact with about 688 genes, comprising several auxin-related genes (Xian et al., 2020). This finding further highlights the key role of TFs in the regulation of HM stress tolerance. In pea seedlings, Cd-induced oxidative stress activated the peroxidase enzymes that lead to auxin oxidation and reduced its stability (Chaoui and El Ferjani, 2005). In barley root tips, it was shown that H_2_O_2_ acts downstream of IAA in root responses to Cd stress. As a consequence, a decrease in auxin concentration triggers a burst in ROS production, repressing the expression of genes involved in cell cycle regulation and root growth (Demecsová et al., 2020).

Another aspect of HMs-induced stress involves the regulation of miRNAs, key factors in modulating auxin homeostasis. Recent findings show that several miRNAs are upregulated by HMs and that they target components of the auxin signalling cascade, such as TIR1, AFBs, and ARFs (Gao et al., 2019; Du et al., 2022). This response involves the regulatory network that controls adventitious rooting, based on the interaction of miR160, 167, and *ARF6, 8*, and *17*. Cd, Al, and As stress often induces deregulation of mRNAs, modulating root elongation, or lateral root formation by acting on auxin responsive gene regulation, signalling and degradation (Ding et al., 2011; Lima et al., 2011; Srivastava et al., 2013; Gao et al., 2019; Kuang et al., 2021; Pegler et al., 2021; Liu et al., 2022). In detail, *AtARF10* and *AtARF16* are targeted by miR160, which controls the development of the root cap (Wang et al., 2005; Khan et al., 2011), while miR390 has been reported to control the generation of tasiRNA (TAS3-derived trans-acting short-interfering RNA), which regulates lateral root growth by targeting transcription factors such as *AtARF2, AtARF3* and *AtARF4* (Marin et al., 2010; Yoon et al., 2010). miR528 targets IAA-alanine resistance protein 1 (*IAR1*), which is involved in the regulation of free cellular auxin, leading to an SCF-mediated protein increase (Li et al., 2019). In addition, the TIR1-F-box subunit is targeted by miR393 upon HMs stress.

**FIGURE 8:**
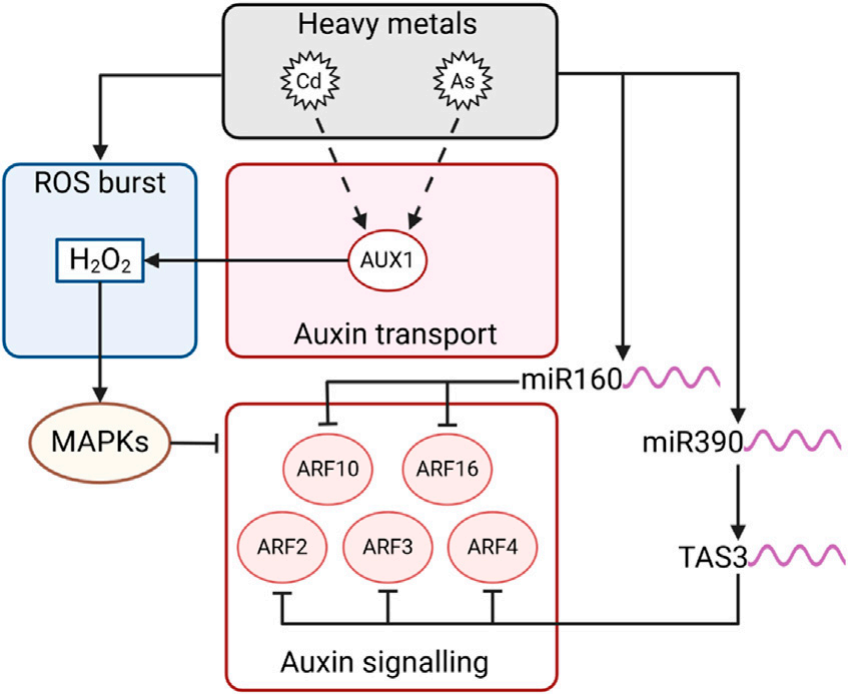
Schematic representation of the interplay among auxin signalling and ROS upon HMs stress. Arrows indicate positive regulation, and T-bars indicate negative regulation. Dashed lines represent multiple steps or unconfirmed direct interactions.

Altogether these data underline that the fine tuning existing between auxin and ROS during HMs responses is obtained via different pathways (Figure 8).

## 4 Concluding remarks and future perspectives

Despite the recent progress in the molecular mechanisms employed by plants to face abiotic stresses, further studies are needed to fully clarify the role of auxin in these processes. It is still not clear, for example, whether variation in auxin dynamics might be coordinators of stress response or whether those are mere consequences of the activation of other pathways such as the ABA one. However, up to date, it is clear that the alteration of auxin dynamics in response to stresses results in the overall alteration of plant growth. Future studies will permit to define the exact role of auxin in response to stress.

**FIGURE 9:**
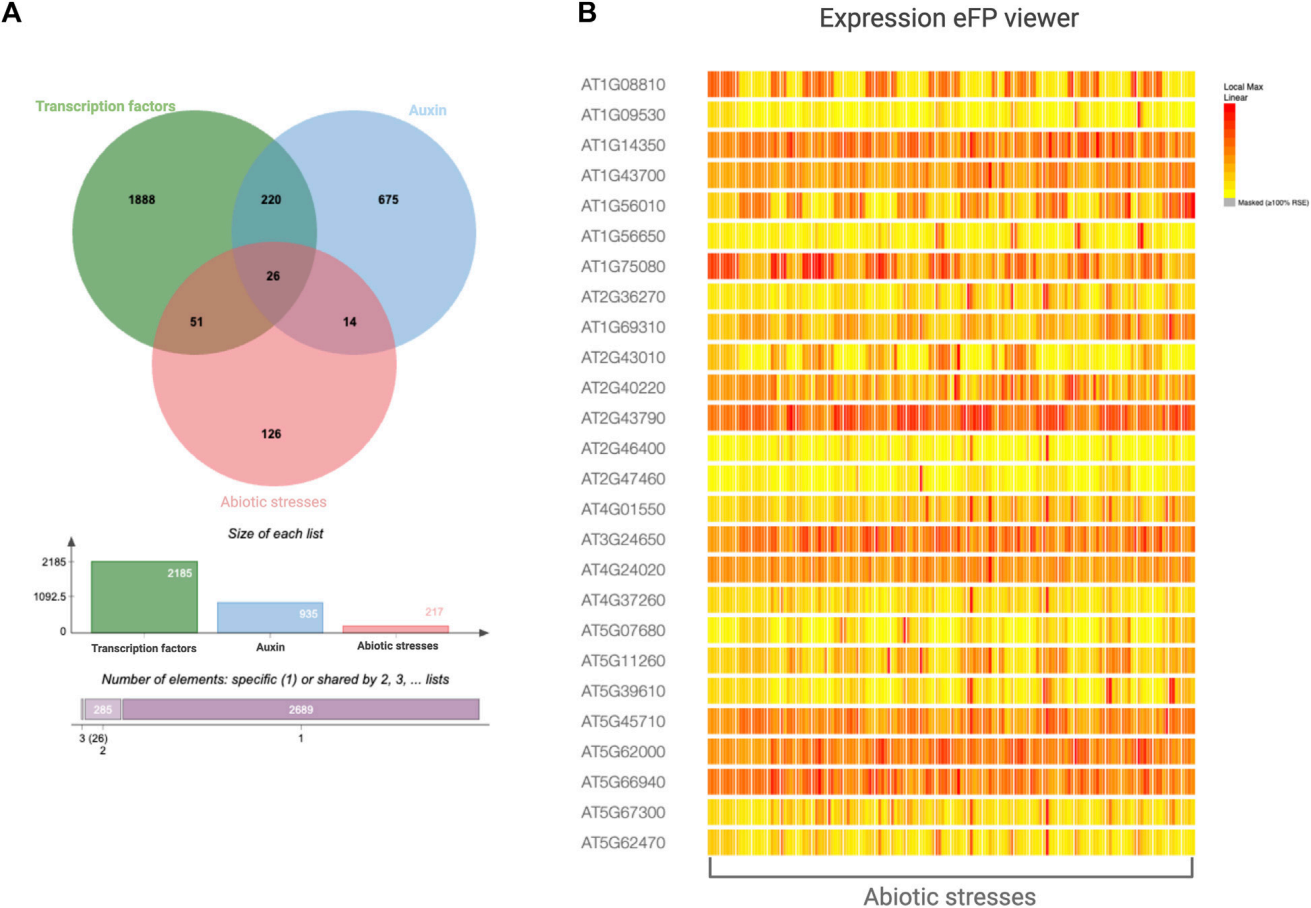
Transcription factors, auxin-related, and abiotic stress-related genes reported in the NCBI Gene database of *Arabidopsis*. **(A)** Venn diagram reporting the intersection among genes annotated as transcription factors, auxin-related, and abiotic stress-related. **(B)** The eFP viewer expression pattern of the core genes (26) depicted in the Venn diagram and reported in Supplementary Table S1.

Many transcription factors in Arabidopsis have been linked to auxin or abiotic challenges, but only a few have been demonstrated to directly regulate auxin dynamics in response to abiotic stimuli, such as drought and salt stress (Table 1). We searched the NCBI gene database of Arabidopsis using the keywords ‘transcription factor’, ‘auxin’, and ‘abiotic stress’. By analysing the overlapping data sets, we identified 26 transcription factors that were associated with all the searched terms (Figure 9A). Their transcriptional level largely varies across the different abiotic stresses (Figure 9B; Supplementary Table S1). We believe that these promising transcription factors are of primary interest for future research aimed at investigating their involvement in modulating auxin responses to abiotic stressors in both model and crop plant species.

**TABLE 1:** Most relevant TFs reported to be related to auxin regulation in response to abiotic stresses.

Gene name	Plant species	Function	Regulation by miRNA	Abiotic stress	Reference
*ARF2*	*Solanum lycopersicum*; *Arabidopsis thaliana*	Regulation of auxin signalling	miR390	Salt, drought, heavy metals	Bouzroud et al. (2018), El Mamoun et al. (2023), Montgomery et al. (2008), Marin et al. (2010)
*ARF3*	*Arabidopsis thaliana*	Regulation of auxin signalling	miR390	Salt, drought, heavy metals	Montgomery et al. (2008), Marin et al. (2010), Yoon et al. (2010)
*ARF4*	*Solanum lycopersicum*; *Arabidopsis thaliana*	Regulation of auxin signalling	miR390	Salt, drought, heavy metals	Bouzroud et al. (2018), Bouzroud et al. (2020) Chen et al. (2021), Montgomery et al. (2008), Marin et al. (2010), Yoon et al. (2010)
*ARF6*	*Solanum lycopersicum*; *Arabidopsis thaliana*	Regulation of auxin signalling	miR167	Salt, drought, heavy metals	Montgomery et al. (2008), Marin et al. (2010), Wang et al. (2005), Gutierrez et al. (2009)
*ARF8*	*Solanum lycopersicum*; *Arabidopsis thaliana*	Regulation of auxin signalling	miR167	Salt, drought, heavy metals	Montgomery et al. (2008), Marin et al. (2010), Wang et al. (2005), Gutierrez et al. (2009)
*ARF10*	*Arabidopsis thaliana*	Regulation of auxin signalling	miR160	Salt, drought	Montgomery et al. (2008), Liu et al. (2010), Wang et al. (2005)
*ARF16*	*Arabidopsis thaliana*	Regulation of auxin signalling	miR160	Salt, drought	Montgomery et al., 2008; Liu et al. (2010), Wang et al. (2005)
*ARF17*	*Arabidopsis thaliana*	Regulation of auxin signalling	miR160	Salt, drought, heavy metals	Montgomery et al. (2008), Gutierrez et al. (2009); Liu et al. (2010)
*MADS25*	*Oryza sativa*; *Poa pratensis*	Induction of auxin biosynthesis; interaction with auxin-related genes	-	Salt, heavy metals	Xu et al. (2018)
*ABO3*	*Arabidopsis thaliana*	Responsive to auxin signalling	-	Drought	Salehin et al. (2019)
*RAB21*	*Oryza sativa*	Responsive to auxin signalling	-	Drought	Zhang et al. (2021)
*CBF/DREBs*	*Arabidopsis thaliana*	Regulation of auxin signalling	-	Drought	Shani et al. (2017)
*NAC20*	*Glycine max*	Regulation of auxin signalling	-	Salt	Shao et al. (2015)
*NAC11*	*Glycine max*	Regulation of auxin signalling	-	Salt	Shao et al. (2015)
*NAC46*	*Arabidopsis thaliana*	Regulation of auxin signalling	-	Salt, drought	Ma et al. (2021)
*WRKY3*	*Solanum lycopersicum*	Regulation of auxin transport	-	Salt	Hichri et al. (2017)
*EIN3*	*Arabidopsis thaliana*	Regulation of auxin biosynthesis	-	Heavy metals	Yang et al. (2019), Liu et al. (2016)

Understanding the complex network of transcription factors regulating auxin biosynthesis and signalling could help find new biotechnological approaches to deal with adverse environmental conditions, hopefully leading to the identification of useful traits that might improve plant adaptation and resilience to GCC. Besides, modulation of miRNAs to alter ARF abundance during abiotic stress and the integration of new technologies such as CRISPR to edit auxin-related genes in crop species are also potential agricultural applications for improving stress tolerance. The use of different research tools such as expression profiling, mutant screening, microarrays, proteomics, system biology, and bioinformatics can offer a powerful platform for the identification as well as characterization of various molecular pathways involved in auxin-mediated HMs stress tolerance and cross-talk with other phytohormones and signalling molecules as ROS in plants.

## References

[B1] Ahanger M. A. Tomar N. S. Tittal M. Argal S. Agarwal R. M. (2017). Plant growth under water/salt stress: ROS production; antioxidants and significance of added potassium under such conditions. Physiol. Mol. Biol. Plants 23, 731–744. 10.1007/s12298-017-0462-7 29158624 PMC5671444

[B2] Ali B. Gill R. A. (2022). Editorial: heavy metal toxicity in plants: recent insights on physiological and molecular aspects, volume II. Front. Plant Sci. 13, 1016257. 10.3389/fpls.2022.1016257 36340414 PMC9634536

[B3] Ashraf M. A. Umetsu K. Ponomarenko O. Saito M. Aslam M. Antipova O. (2020). PIN FORMED 2 modulates the transport of arsenite in *Arabidopsis thaliana* . Plant Commun. 1, 100009. 10.1016/j.xplc.2019.100009 33404549 PMC7747963

[B4] Bai B. Bian H. Zeng Z. Hou N. Shi B. Wang J. (2017). MiR393-Mediated auxin signaling regulation is involved in root elongation inhibition in response to toxic aluminum stress in barley. Plant Cell Physiol. 58, 426–439. 10.1093/pcp/pcw211 28064248

[B5] Bernatchez L. Ferchaud A. L. Berger C. S. Venney C. J. Xuereb A. (2023). Genomics for monitoring and understanding species responses to global climate change. Nat. Rev. Genet. 25, 165–183. 10.1038/s41576-023-00657-y 37863940

[B6] Bertolotti G. Scintu D. Dello Ioio R. (2021). A small cog in a large wheel: crucial role of miRNAs in root apical meristem patterning. J. Exp. Bot. 72, 6755–6767. 10.1093/jxb/erab332 34350947

[B7] Blilou I. Xu J. Wildwater M. Willemsen V. Paponov I. Friml J. (2005). The PIN auxin efflux facilitator network controls growth and patterning in Arabidopsis roots. Nature 433, 39–44. 10.1038/nature03184 15635403

[B8] Bouzroud S. Gasparini K. Hu G. Barbosa M. A. M. Rosa B. L. Fahr M. (2020). Down regulation and loss of auxin response factor 4 function using CRISPR/Cas9 alters plant growth, stomatal function and improves tomato tolerance to salinity and osmotic stress. Genes 11 (3), 272. 10.3390/genes11030272 32138192 PMC7140898

[B9] Bouzroud S. Gouiaa S. Hu N. Bernadac A. Mila I. Bendaou N. (2018). Auxin response factors (ARFs) are potential mediators of auxin action in tomato response to biotic and abiotic stress (Solanum lycopersicum). PLoS ONE 13 (2), e0193517. 10.1371/journal.pone.0193517 29489914 PMC5831009

[B10] Cackett L. Cannistraci C. V. Meier S. Ferrandi P. Pěnčík A. Gehring C. (2022). Salt-specific gene expression reveals elevated auxin levels in *Arabidopsis thaliana* plants grown under saline conditions. Front. Plant Sci. 13, 804716. 10.3389/fpls.2022.804716 35222469 PMC8866861

[B11] Calabrese E. J. Dhawan G. Kapoor R. Iavicoli I. Calabrese V. (2016). HORMESIS: a fundamental concept with widespread biological and biomedical applications. Gerontology 62 (5), 530–535. 10.1159/000441520 26535577

[B12] Cao X. Yang H. Shang C. Ma S. Liu L. Cheng J. (2019). The roles of auxin biosynthesis YUCCA gene family in plants. Int. J. Mol. Sci. 20, 6343–6410. 10.3390/ijms20246343 31888214 PMC6941117

[B13] Cavallari N. Artner C. Benkova E. (2021). Auxin-regulated lateral root organogenesis. Cold Spring Harb. Perspect. Biol. 13 (7), a039941. 10.1101/cshperspect.a039941 33558367 PMC8247565

[B14] Cha J.-Y. Jeong S. Y. Ahn G. Shin G.-I. Ji M. G. Lee S. C. (2022). The thiol-reductase activity of YUCCA6 enhances nickel heavy metal stress tolerance in *Arabidopsis* . Front. Plant Sci. 13, 1007542. 10.3389/fpls.2022.1007542 36237515 PMC9551240

[B15] Chaoui A. El Ferjani E. (2005). Effects of cadmium and copper on antioxidant capacities, lignification and auxin degradation in leaves of pea (Pisum sativum L.) seedlings. Comptes Rendus - Biol. 328, 23–31. 10.1016/j.crvi.2004.10.001 15714877

[B16] Chen M. Zhu X. Liu X. Wu C. Yu C. Hu G. (2021). Knockout of auxin response factor SlARF4 improves tomato resistance to water deficit. Int. J. Mol. Sci. 22 (7), 3347. 10.3390/ijms22073347 33805879 PMC8037468

[B17] Chen Z. Hu L. Han N. Hu J. Yang Y. Xiang T. (2015). Overexpression of a miR393-resistant form of transport inhibitor response protein 1 (mTIR1) enhances salt tolerance by increased osmoregulation and Na^+^ exclusion in *Arabidopsis thaliana* . Plant Cell Physiol. 56, 73–83. 10.1093/pcp/pcu149 25336111

[B18] Chen Z. H. Bao M. L. Sun Y. Z. Yang Y. J. Xu X. H. Wang J. H. (2011). Regulation of auxin response by miR393-targeted transport inhibitor response protein 1 is involved in normal development in Arabidopsis. Plant Mol. Biol. 77 (6), 619–629. 10.1007/s11103-011-9838-1 22042293

[B19] Cramer G. R. Urano K. Delrot S. Pezzotti M. Shinozaki K. (2011). Effects of abiotic stress on plants: a systems biology perspective. BMC Plant Biol. 11, 163. 10.1186/1471-2229-11-163 22094046 PMC3252258

[B20] Cruz de Carvalho M. H. (2008). Drought stress and reactive oxygen species: production, scavenging and signaling. Plant Signal. Behav. 3 (3), 156–165. 10.4161/psb.3.3.5536 19513210 PMC2634109

[B21] Dat J. Vandenabeele S. Vranová E. Van Montagu M. Inzé D. Van Breusegem F. (2000). Dual action of the active oxygen species during plant stress responses. Cell. Mol. Life Sci. 57, 779–795. 10.1007/s000180050041 10892343 PMC11147059

[B22] Dello Ioio R. Galinha C. Fletcher A. G. Grigg S. P. Molnar A. Willemsen V. (2012). A PHABULOSA/cytokinin feedback loop controls root growth in Arabidopsis. Curr. Biol. CB 22 (18), 1699–1704. 10.1016/j.cub.2012.07.005 22902752

[B23] Dello Ioio R. Nakamura K. Moubayidin L. Perilli S. Taniguchi M. Morita M. T. (2008). A genetic framework for the control of cell division and differentiation in the root meristem. Science 322 (5906), 1380–1384. 10.1126/science.1164147 19039136

[B24] Demecsová L. Zelinová V. Liptáková Ľ. Tamás L. (2020). Mild cadmium stress induces auxin synthesis and accumulation, while severe cadmium stress causes its rapid depletion in barley root tip. Environ. Exp. Bot. 175, 104038. 10.1016/j.envexpbot.2020.104038

[B25] De Smet I. Signora L. Beeckman T. Inzé D. Foyer C. H. Zhang H. (2003). An abscisic acid-sensitive checkpoint in lateral root development of Arabidopsis. Plant J. Cell Mol. Biol. 33 (3), 543–555. 10.1046/j.1365-313x.2003.01652.x 12581312

[B26] Di Mambro R. De Ruvo M. Pacifici E. Salvi E. Sozzani R. Benfey P. N. (2017). Auxin minimum triggers the developmental switch from cell division to cell differentiation in the Arabidopsis root. Proc. Natl. Acad. Sci. U. S. A. 114 (36), E7641–E7649. 10.1073/pnas.1705833114 28831001 PMC5594665

[B27] Di Mambro R. Sabatini S. Dello Ioio R. (2018). Patterning the axes: a lesson from the root. Plants Basel, Switz. 8 (1), 8. 10.3390/plants8010008 PMC6358898 30602700

[B28] Ding Y. Chen Z. Zhu C. (2011). Microarray-based analysis of cadmium-responsive microRNAs in rice (Oryza sativa). J. Exp. Bot. 62, 3563–3573. 10.1093/jxb/err046 21362738 PMC3130178

[B29] Dong C. Ma Y. Wisniewski M. Cheng Z. M. (2017). Meta-analysis of the effect of overexpression of CBF/DREB family genes on drought stress response. Environ. Exp. Bot. 142, 1–14. 10.1016/j.envexpbot.2017.07.014

[B30] Du W. Lu Y. Li Q. Luo S. Shen S. Li N. (2022). TIR1/AFB proteins: active players in abiotic and biotic stress signaling. Front. Plant Sci. 13, 1083409–1083413. 10.3389/fpls.2022.1083409 36523629 PMC9745157

[B31] Dubey S. Saxena S. Chauhan A. S. Mathur P. Rani V. Chakrabaroty D. (2020). Identification and expression analysis of conserved microRNAs during short and prolonged chromium stress in rice (Oryza sativa). Environ. Sci. Pollut. Res. Int. 27 (1), 380–390. 10.1007/s11356-019-06760-0 31792790

[B32] El Mamoun I. Bouzroud S. Zouine M. Smouni A. (2023). The knockdown of AUXIN RESPONSE FACTOR 2 confers enhanced tolerance to salt and drought stresses in tomato (Solanum lycopersicum L). Plants 12 (15), 2804. 10.3390/plants12152804 37570958 PMC10420960

[B33] Fattorini L. Ronzan M. Piacentini D. Della Rovere F. De Virgilio C. Sofo A. (2017). Cadmium and arsenic affect quiescent centre formation and maintenance in *Arabidopsis thaliana* post-embryonic roots disrupting auxin biosynthesis and transport. Environ. Exp. Bot. 144, 37–48. 10.1016/j.envexpbot.2017.10.005

[B34] Finnegan P. M. Chen W. (2012). Arsenic toxicity: the effects on plant metabolism. Front. Physiol. 3, 182. 10.3389/fphys.2012.00182 22685440 PMC3368394

[B35] Friml J. Gallei M. Gelová Z. Johnson A. Mazur E. Monzer A. (2022). ABP1–TMK auxin perception for global phosphorylation and auxin canalization. Nature 609 (7927), 575–581. 10.1038/s41586-022-05187-x 36071161

[B36] Fu Y. Yang Y. Chen S. Ning N. Hu H. (2019). Arabidopsis IAR4 modulates primary root growth under salt stress through ros-mediated modulation of auxin distribution. Front. Plant Sci. 10, 522. 10.3389/fpls.2019.00522 31105724 PMC6494962

[B37] Gao J. Luo M. Peng H. Chen F. Li W. (2019). Characterization of cadmium-responsive MicroRNAs and their target genes in maize (Zea mays) roots. BMC Mol. Biol. 20, 14. 10.1186/s12867-019-0131-1 31046674 PMC6498490

[B38] Geng Y. Wu R. Wee C. W. Xie F. Wei X. Chan P. M. Y. (2013). A spatio-temporal understanding of growth regulation during the salt stress response in Arabidopsis. Plant Cell 25, 2132–2154. 10.1105/tpc.113.112896 23898029 PMC3723617

[B39] Ghelli R. Brunetti P. Marzi D. Cecchetti V. Costantini M. Lanzoni-Rossi M. (2023). The full-length Auxin Response Factor 8 isoform ARF8.1 controls pollen cell wall formation and directly regulates TDF1, AMS and MS188 expression. Plant J. 113 (4), 851–865. 10.1111/tpj.16089 36597651

[B40] Gutierrez L. Bussell J. D. Pǎcurar D. I. Schwambach J. Pǎcurar M. Bellini C. (2009). Phenotypic plasticity of adventitious rooting in arabidopsis is controlled by complex regulation of AUXIN RESPONSE FACTOR transcripts and microRNA abundance. Plant Cell 21, 3119–3132. 10.1105/tpc.108.064758 19820192 PMC2782293

[B41] Hao K. Wang Y. Zhu Z. Wu Y. Chen R. Zhang L. (2022). miR160: an indispensable regulator in plant. Front. Plant Sci. 13, 833322. 10.3389/fpls.2022.833322 35392506 PMC8981303

[B42] Hao R. Zhou W. Li J. Luo M. Scheres B. Guo Y. (2023). On salt stress, PLETHORA signaling maintains root meristems. Dev. Cell 58 (18), 1657–1669.e5. 10.1016/j.devcel.2023.06.012 37480843

[B43] Hao Y. J. Wei W. Song Q. X. Chen H. W. Zhang Y. Q. Wang F. (2011). Soybean NAC transcription factors promote abiotic stress tolerance and lateral root formation in transgenic plants. Plant J. 68 (2), 302–313. 10.1111/j.1365-313X.2011.04687.x 21707801

[B44] Hasanuzzaman M. Bhuyan MHMB Zulfiqar F. Raza A. Mohsin S. M. Al Mahmud J. (2020). Reactive oxygen species and antioxidant defense in plants under abiotic stress: revisiting the crucial role of a universal defense regulator. Antioxidants 9, 681–752. 10.3390/antiox9080681 32751256 PMC7465626

[B45] Hassan M. U. Chattha M. U. Khan I. Chattha M. B. Aamer M. Nawaz M. (2019). Nickel toxicity in plants: reasons, toxic effects, tolerance mechanisms, and remediation possibilities—a review. Environ. Sci. Pollut. Res. 26, 12673–12688. 10.1007/s11356-019-04892-x 30924044

[B46] Hayat S. Hayat Q. Alyemeni M. N. Wani A. S. Pichtel J. Ahmad A. (2012). Role of proline under changing environments: a review. Plant Signal. Behav. 7 (Issue 11), 1456–1466. 10.4161/psb.21949 22951402 PMC3548871

[B47] He X. J. Mu R. L. Cao W. H. Zhang Z. G. Zhang J. S. Chen S. Y. (2005). AtNAC2, a transcription factor downstream of ethylene and auxin signaling pathways, is involved in salt stress response and lateral root development. Plant J. 44 (6), 903–916. 10.1111/j.1365-313X.2005.02575.x 16359384

[B48] Hichri I. Muhovski Y. Žižková E. Dobrev P. I. Gharbi E. Franco-Zorrilla J. M. (2017). The *Solanum lycopersicum* WRKY3 transcription factor SlWRKY3 is involved in salt stress tolerance in tomato. Front. Plant Sci. 8, 1343. 10.3389/fpls.2017.01343 28824679 PMC5534461

[B49] Hu Y. F. Zhou G. Na X. F. Yang L. Nan W. Liu X. (2013). Cadmium interferes with maintenance of auxin homeostasis in Arabidopsis seedlings. J. Plant Physiol. 170, 965–975. 10.1016/j.jplph.2013.02.008 23683587

[B50] Huang J. Li Z. Zhao D. (2016). Deregulation of the OsmiR160 target gene OsARF18 causes growth and developmental defects with an alteration of auxin signaling in rice. Sci. Rep. 6, 29938. 10.1038/srep29938 27444058 PMC4956771

[B51] Jia X. L. Li M. Y. Jiang Q. Xu Z. S. Wang F. Xiong A. S. (2015). High-throughput sequencing of small RNAs and anatomical characteristics associated with leaf development in celery. Sci. Rep. 5, 11093. 10.1038/srep11093 26057455 PMC4460894

[B52] Jiang Y. Deyholos M. K. (2006). Comprehensive transcriptional profiling of NaCl-stressed Arabidopsis roots reveals novel classes of responsive genes. BMC plant Biol. 6, 25. 10.1186/1471-2229-6-25 17038189 PMC1621065

[B53] Jung H. Lee D. K. Choi Y.Do Kim J. K. (2015). OsIAA6, a member of the rice Aux/IAA gene family, is involved in drought tolerance and tiller outgrowth. Plant Sci. 236, 304–312. 10.1016/j.plantsci.2015.04.018 26025543

[B54] Kalve S. Sizani B. L. Markakis M. N. Helsmoortel C. Vandeweyer G. Laukens K. (2020). Osmotic stress inhibits leaf growth of *Arabidopsis thaliana* by enhancing ARF-mediated auxin responses. New Phytol. 226 (6), 1766–1780. 10.1111/nph.16490 32077108

[B55] Kaur H. Garg N. (2021). Zinc toxicity in plants: a review. Planta 253, 129. 10.1007/s00425-021-03642-z 34043068

[B56] Ke Q. Wang Z. Ji C. Y. Jeong J. C. Lee H. S. Li H. (2015). Transgenic poplar expressing Arabidopsis YUCCA6 exhibits auxin-overproduction phenotypes and increased tolerance to abiotic stress. Plant Physiology Biochem. 94, 19–27. 10.1016/j.plaphy.2015.05.003 25980973

[B57] Khan G. A. Declerck M. Sorin C. Hartmann C. Crespi M. Lelandais-Brière C. (2011). MicroRNAs as regulators of root development and architecture. Plant Mol. Biol. 77, 47–58. 10.1007/s11103-011-9793-x 21607657

[B58] Kim J. I. Baek D. Park H. C. Chun H. J. Oh D. H. Lee M. K. (2013). Overexpression of Arabidopsis YUCCA6 in potato results in high-auxin developmental phenotypes and enhanced resistance to water deficit. Mol. Plant 6 (2), 337–349. 10.1093/mp/sss100 22986790

[B59] Kovtun Y. Chiu W.-L. Tena G. Sheen J. (2000). Functional analysis of oxidative stress-activated mitogen-activated protein kinase cascade in plants. Proc. Natl. Acad. Sci. 97, 2940–2945. 10.1073/pnas.97.6.2940 10717008 PMC16034

[B60] Křeček P. Skůpa P. Libus J. Naramoto S. Tejos R. Friml J. (2009). The PIN-FORMED (PIN) protein family of auxin transporters. Genome Biol. 12, 249–311. 10.1186/gb-2009-10-12-249 PMC2812941 20053306

[B61] Krishnamurthy A. Rathinasabapathi B. (2013). Auxin and its transport play a role in plant tolerance to arsenite-induced oxidative stress in *Arabidopsis thaliana* . Plant, Cell Environ. 36, 1838–1849. 10.1111/pce.12093 23489261

[B62] Kuang L. Yu J. Shen Q. Fu L. Wu L. (2021). Identification of micrornas responding to aluminium, cadmium and salt stresses in barley roots. Plants 10, 2754–2814. 10.3390/plants10122754 34961225 PMC8704135

[B63] Kumar A. Verma K. Kashyap R. Joshi V. J. Sircar D. Yadav S. R. (2024). Auxin-responsive ROS homeostasis genes display dynamic expression pattern during rice crown root primordia morphogenesis. Plant Physiol. Biochem. 206, 108307. 10.1016/j.plaphy.2023.108307 38159549

[B64] Kurek K. Plitta-Michalak B. Ratajczak E. (2019). Reactive oxygen species as potential drivers of the seed aging process. Plants 8, 174. 10.3390/plants8060174 31207940 PMC6630744

[B65] Lambert S. A. Jolma A. Campitelli L. F. Das P. K. Yin Y. Albu M. (2018). The human transcription factors. Cell 172 (4), 650–665. 10.1016/j.cell.2018.01.029 29425488 PMC12908702

[B66] Lata C. Prasad M. (2011). Role of DREBs in regulation of abiotic stress responses in plants. J. Exp. Bot. 62 (14), 4731–4748. 10.1093/jxb/err210 21737415

[B67] Lee M. Jung J. H. Han D. Y. Seo P. J. Park W. J. Park C. M. (2012). Activation of a flavin monooxygenase gene YUCCA7 enhances drought resistance in Arabidopsis. Planta 235 (5), 923–938. 10.1007/s00425-011-1552-3 22109847

[B68] Lelli K. M. Slattery M. Mann R. S. (2012). Disentangling the many layers of eukaryotic transcriptional regulation. Annu. Rev. Genet. 46, 43–68. 10.1146/annurev-genet-110711-155437 22934649 PMC4295906

[B69] Lequeux H. Hermans C. Lutts S. Verbruggen N. (2010). Response to copper excess in *Arabidopsis thaliana*: impact on the root system architecture, hormone distribution, lignin accumulation and mineral profile. Plant Physiol. biochem. 48, 673–682. 10.1016/j.plaphy.2010.05.005 20542443

[B70] Lešková A. Zvarĺk M. Araya T. Giehl R. F. H. (2020). Nickel toxicity targets cell wall-related processes and PIN2-mediated auxin transport to inhibit root elongation and gravitropic responses in Arabidopsis. Plant Cell Physiol. 61, 519–535. 10.1093/pcp/pcz217 31750920 PMC7065594

[B71] Leyser O. (2018). Auxin signaling. Plant Physiol. 176, 465–479. 10.1104/pp.17.00765 28818861 PMC5761761

[B72] Li J. Jia Y. Dong R. Huang R. Liu P. Li X. (2019). Advances in the mechanisms of plant tolerance to manganese toxicity. Int. J. Mol. Sci. 20, 5096. 10.3390/ijms20205096 31615142 PMC6834138

[B73] Lima J. C. Arenhart R. A. Margis-Pinheiro M. Margis R. (2011). Aluminum triggers broad changes in microRNA expression in rice roots. Genet. Mol. Res. 10, 2817–2832. 10.4238/2011.November.10.4 22095606

[B74] Lin W. Zhou X. Tang W. Takahashi K. Pan X. Dai J. (2021). TMK-based cell-surface auxin signalling activates cell-wall acidification. Nature 599, 278–282. 10.1038/s41586-021-03976-4 34707287 PMC8549421

[B75] Liu G. Gao S. Tian H. Wu W. Robert H. S. Ding Z. (2016). Local transcriptional control of YUCCA regulates auxin promoted root-growth inhibition in response to aluminium stress in arabidopsis. PLoS Genet. 12, 10063600–e1006425. 10.1371/journal.pgen.1006360 PMC5065128 27716807

[B76] Liu H. Zhu R. Shu K. Lv W. Wang S. Wang C. (2022). Aluminum stress signaling, response, and adaptive mechanisms in plants. Plant Signal. Behav. 17, 2057060. 10.1080/15592324.2022.2057060 35467484 PMC9045826

[B77] Liu W. Li R. J. Han T. T. Cai W. Fu Z. W. Lu Y. T. (2015). Salt stress reduces root meristem size by nitric oxide-mediated modulation of auxin accumulation and signaling in Arabidopsis. Plant physiol. 168 (1), 343–356. 10.1104/pp.15.00030 25818700 PMC4424022

[B78] Liu X. Huang J. Wang Y. Khanna K. Xie Z. Owen H. A. (2010). The role of floral organs in carpels, an Arabidopsis loss-of-function mutation in MicroRNA160a, in organogenesis and the mechanism regulating its expression. Plant J. 62, 416–428. 10.1111/j.1365-313X.2010.04164.x 20136729

[B79] Lu C. Chen M. X. Liu R. Zhang L. Hou X. Liu S. (2019). Abscisic acid regulates auxin distribution to mediate maize lateral root development under salt stress. Front. Plant Sci. 10, 716–16. 10.3389/fpls.2019.00716 31231407 PMC6560076

[B80] Luo Y. Wei Y. Sun S. Wang J. Wang W. Han D. (2019). Selenium modulates the level of auxin to alleviate the toxicity of cadmium in tobacco. Int. J. Mol. Sci. 20, 3772. 10.3390/ijms20153772 31374993 PMC6696094

[B81] Ma J. Wang L. Dai J. Wang Y. Lin D. (2021). The NAC-type transcription factor CaNAC46 regulates the salt and drought tolerance of transgenic *Arabidopsis thaliana* . BMC Plant Biol. 21 (1), 11. 10.1186/s12870-020-02764-y 33407148 PMC7788707

[B82] Małkowski E. Sitko K. Szopiński M. Gieroń Ż. Pogrzeba M. Kalaji H. M. (2020). Hormesis in plants: the role of oxidative stress, auxins and photosynthesis in corn treated with Cd or Pb. Int. J. Mol. Sci. 21, 2099. 10.3390/ijms21062099 32204316 PMC7139973

[B83] Marin E. Jouannet V. Herz A. Lokerse A. S. Weijers D. Vaucheret H. (2010). miR390, Arabidopsis TAS3 tasiRNAs, and their AUXIN RESPONSE FACTOR targets define an autoregulatory network quantitatively regulating lateral root growth. Plant Cell 22 (4), 1104–1117. 10.1105/tpc.109.072553 20363771 PMC2879756

[B146] Marzi D. Brunetti P. Mele G. Napoli N. Calò L. Spaziani E. (2020). Light controls stamen elongation via cryptochromes, phytochromes and COP1 through HY5 and HYH. Plant J. 103, 379–394. 10.1111/tpj.14736 32142184

[B84] Meng L. S. Wang Z. B. Yao S. Q. Liu A. (2015). The ARF2-ANT-COR15A gene cascade regulates ABA-signaling-mediated resistance of large seeds to drought in Arabidopsis. J. Cell Sci. 128 (21), 3922–3932. 10.1242/jcs.171207 26395398

[B85] Mickelbart M. V. Hasegawa P. M. Bailey-Serres J. (2015). Genetic mechanisms of abiotic stress tolerance that translate to crop yield stability. Nat. Rev. Genet. 16, 237–251. 10.1038/nrg3901 25752530

[B86] Montgomery T. A. Howell M. D. Cuperus J. T. Li D. Hansen J. E. Alexander A. L. (2008). Specificity of ARGONAUTE7-miR390 interaction and dual functionality in TAS3 trans-acting siRNA formation. Cell 133 (1), 128–141. 10.1016/j.cell.2008.02.033 18342362

[B87] Müller C. J. Valdés A. E. Wang G. Ramachandran P. Beste L. Uddenberg D. (2016). PHABULOSA mediates an auxin signaling loop to regulate vascular patterning in Arabidopsis. Plant Physiol. 170, 956–970. 10.1104/pp.15.01204 26637548 PMC4734557

[B88] Niu C. F. Wei W. Zhou Q. Y. Tian A. G. Hao Y. J. Zhang W. K. (2012). Wheat WRKY genes TaWRKY2 and TaWRKY19 regulate abiotic stress tolerance in transgenic Arabidopsis plants. Plant, Cell and Environ. 35 (6), 1156–1170. 10.1111/j.1365-3040.2012.02480.x 22220579

[B89] Nodzyński T. Vanneste S. Zwiewka M. Pernisová M. Hejátko J. Friml J. (2016). Enquiry into the topology of plasma membrane-localized PIN auxin transport components. Mol. plant 9 (11), 1504–1519. 10.1016/j.molp.2016.08.010 27622590 PMC5106287

[B90] Overvoorde P. Fukaki H. Beeckman T. (2010). Auxin control of root development. Cold Spring Harb. Perspect. Biol. 2, a001537. 10.1101/cshperspect.a001537 20516130 PMC2869515

[B91] Pacifici E. Di Mambro R. Dello Ioio R. Costantino P. Sabatini S. (2018). Acidic cell elongation drives cell differentiation in the Arabidopsis root. EMBO J. 37, e99134–e99139. 10.15252/embj.201899134 30012836 PMC6092616

[B92] Pegler J. L. Oultram J. M. J. Nguyen D. Q. Grof C. P. L. Eamens A. L. (2021). Microrna-mediated responses to cadmium stress in arabidopsis thaliana. Plants 10, 130–224. 10.3390/plants10010130 33435199 PMC7827075

[B93] Powers S. K. Strader L. C. (2020). Regulation of auxin transcriptional responses. Dev. Dyn. 249, 483–495. 10.1002/dvdy.139 31774605 PMC7187202

[B94] Praveen A. Pandey A. Gupta M. (2019). Nitric oxide alters nitrogen metabolism and PIN gene expressions by playing protective role in arsenic challenged Brassica juncea L. Ecotoxicol. Environ. Saf. 176, 95–107. 10.1016/j.ecoenv.2019.03.054 30925332

[B95] Promchuea S. Zhu Y. Chen Z. Zhang J. Gong Z. (2017). ARF2 coordinates with PLETHORAs and PINs to orchestrate ABA-mediated root meristem activity in Arabidopsis. J. Integr. plant Biol. 59 (1), 30–43. 10.1111/jipb.12506 28074634

[B96] Puglia G. D. (2023). Reactive oxygen and nitrogen species (RONS) signalling in seed dormancy release, perception of environmental cues, and heat stress response. Plant Growth Regul. 2023, 01094. 10.1007/s10725-023-01094-x

[B97] Ramachandran P. Wang G. Augstein F. De Vries J. Carlsbecker A. (2018). Continuous root xylem formation and vascular acclimation to water deficit involves endodermal ABA signalling via miR165. Dev 145, dev159202–7. 10.1242/dev.159202 29361572

[B98] Reinhart B. J. Weinstein E. G. Rhoades M. W. Bartel B. Bartel D. P. (2002). MicroRNAs in plants. Genes Dev. 16, 1616–1626. 10.1101/gad.1004402 12101121 PMC186362

[B99] Ribba T. Garrido-Vargas F. O’Brien J. A. (2020). Auxin-mediated responses under salt stress: from developmental regulation to biotechnological applications. J. Exp. Bot. 71, 3843–3853. 10.1093/jxb/eraa241 32433743

[B100] Ronzan M. Piacentini D. Fattorini L. Della Rovere F. Eiche E. Riemann M. (2018). Cadmium and arsenic affect root development in Oryza sativa L. negatively interacting with auxin. Environ. Exp. Bot. 151, 64–75. 10.1016/j.envexpbot.2018.04.008

[B101] Sabatini S. Beis D. Wolkenfelt H. Murfett J. Guilfoyle T. Malamy J. (1999). An auxin-dependent distal organizer of pattern and polarity in the arabidopsis root. Cell 99, 463–472. 10.1016/s0092-8674(00)81535-4 10589675

[B102] Salehin M. Li B. Tang M. Katz E. Song L. Ecker J. R. (2019). Auxin-sensitive Aux/IAA proteins mediate drought tolerance in Arabidopsis by regulating glucosinolate levels. Nat. Commun. 10 (1), 4021. 10.1038/s41467-019-12002-1 31492889 PMC6731224

[B103] Sato A. Soeno K. Kikuchi R. Narukawa-Nara M. Yamazaki C. Kakei Y. (2022). Indole-3-pyruvic acid regulates TAA1 activity, which plays a key role in coordinating the two steps of auxin biosynthesis. Proc. Natl. Acad. Sci. U. S. A. 119 (25), e2203633119. 10.1073/pnas.2203633119 35696560 PMC9231625

[B104] Scintu D. Scacchi E. Cazzaniga F. Vinciarelli F. De Vivo M. Shtin M. (2023). microRNA165 and 166 modulate response of the Arabidopsis root apical meristem to salt stress. Commun. Biol. 6, 834–910. 10.1038/s42003-023-05201-6 37567954 PMC10421904

[B105] Shahid M. Pourrut B. Dumat C. Nadeem M. Aslam M. Pinelli E. (2014). Heavy-metal-induced reactive oxygen species: phytotoxicity and physicochemical changes in plants. Rev. Environ. Contam. Toxicol. 232, 1–44. 10.1007/978-3-319-06746-9_1 24984833

[B106] Shani E. Salehin M. Zhang Y. Sanchez S. E. Doherty C. Wang R. (2017). Plant stress tolerance requires auxin-sensitive aux/IAA transcriptional repressors. Curr. Biol. 27 (3), 437–444. 10.1016/j.cub.2016.12.016 28111153 PMC5296222

[B107] Shao H. Wang H. Tang X. (2015). NAC transcription factors in plant multiple abiotic stress responses: progress and prospects. Front. Plant Sci. 6 (OCTOBER), 902. 10.3389/fpls.2015.00902 26579152 PMC4625045

[B108] Shi H. Chen L. Ye T. Liu X. Ding K. Chan Z. (2014). Modulation of auxin content in Arabidopsis confers improved drought stress resistance. Plant Physiology Biochem. 82, 209–217. 10.1016/j.plaphy.2014.06.008 24992887

[B109] Smolko A. Bauer N. Pavlović I. Pěnčík A. Novák O. Salopek-Sondi B. (2021). Altered root growth, auxin metabolism and distribution in *Arabidopsis thaliana* exposed to salt and osmotic stress. Int. J. Mol. Sci. 22 (15), 7993. 10.3390/ijms22157993 34360759 PMC8348202

[B110] Sofo A. Bochicchio R. Amato M. Rendina N. Vitti A. Nuzzaci M. (2017). Plant architecture, auxin homeostasis and phenol content in *Arabidopsis thaliana* grown in cadmium- and zinc-enriched media. J. Plant Physiol. 216, 174–180. 10.1016/j.jplph.2017.06.008 28704702

[B111] Song H. Wang P. Lin J. Y. Zhao C. Bi Y. Wang X. (2016). Genome-wide identification and characterization of WRKY gene family in peanut. Front. plant Sci. 7, 534. 10.3389/fpls.2016.00534 27200012 PMC4845656

[B112] Srivastava S. Srivastava A. K. Suprasanna P. D’Souza S. F. (2013). Identification and profiling of arsenic stress-induced microRNAs in Brassica juncea. J. Exp. Bot. 64, 303–315. 10.1093/jxb/ers333 23162117

[B113] Stirling E. Fitzpatrick R. W. Mosley L. M. (2020). Drought effects on wet soils in inland wetlands and peatlands. Earth-Science Rev. 210, 103387. 10.1016/j.earscirev.2020.103387

[B114] Sun C. Lu L. Liu L. Liu W. Yu Y. Liu X. (2014). Nitrate reductase-mediated early nitric oxide burst alleviates oxidative damage induced by aluminum through enhancement of antioxidant defenses in roots of wheat (*Triticum aestivum*). New Phytol. 201, 1240–1250. 10.1111/nph.12597 24237306

[B115] Swarup R. Bhosale R. (2019). Developmental roles of AUX1/LAX auxin influx carriers in plants. Front. plant Sci. 10, 1306. 10.3389/fpls.2019.01306 31719828 PMC6827439

[B116] Terrile M. C. París R. Calderõn-Villalobos L. I. A. Iglesias M. J. Lamattina L. Estelle M. (2012). Nitric oxide influences auxin signaling through S-nitrosylation of the Arabidopsis TRANSPORT INHIBITOR RESPONSE 1 auxin receptor. Plant J. 70, 492–500. 10.1111/j.1365-313X.2011.04885.x 22171938 PMC3324642

[B117] Tipping E. Smith E. J. Lawlor A. J. Hughes S. Stevens P. A. (2003). Predicting the release of metals from ombrotrophic peat due to drought-induced acidification. Environ. Pollut. 123, 239–253. 10.1016/S0269-7491(02)00375-5 12628203

[B118] Van Der Woude L. C. Perrella G. Snoek B. L. Van Hoogdalem M. Novák O. Van Verk M. C. (2019). HISTONE DEACETYLASE 9 stimulates auxin-dependent thermomorphogenesis in *Arabidopsis thaliana* by mediating H2A.Z depletion. Proc. Natl. Acad. Sci. U. S. A. 116, 25343–25354. 10.1073/pnas.1911694116 31767749 PMC6911240

[B119] Verma S. Negi N. P. Pareek S. Mudgal G. Kumar D. (2022). Auxin response factors in plant adaptation to drought and salinity stress. Physiol. Plant. 174, e13714. 10.1111/ppl.13714 35560231

[B120] Wang F. Niu H. Xin D. Long Y. Wang G. Liu Z. (2021a). OsIAA18, an aux/IAA transcription factor gene, is involved in salt and drought tolerance in rice. Front. Plant Sci. 12, 738660. 10.3389/fpls.2021.738660 34868122 PMC8637529

[B121] Wang J. Moeen-ud-din M. Yang S. (2021b). Dose-dependent responses of *Arabidopsis thaliana* to zinc are mediated by auxin homeostasis and transport. Environ. Exp. Bot. 189, 104554. 10.1016/j.envexpbot.2021.104554

[B122] Wang J. W. Wang L. J. Mao Y. B. Cai W. J. Xue H. W. Chen X. Y. (2005). Control of root cap formation by MicroRNA-targeted auxin response factors in Arabidopsis. Plant Cell 17, 2204–2216. 10.1105/tpc.105.033076 16006581 PMC1182483

[B123] Wang M. Qiao J. Y. Yu C. L. Chen H. Sun C. D. Huang L. Z. (2019). The auxin influx carrier, OsAUX3, regulates rice root development and responses to aluminium stress. Plant Cell Environ. 42, 1125–1138. 10.1111/pce.13478 30399648

[B124] Wang R. Wang J. Zhao L. Yang S. Song Y. (2015). Impact of heavy metal stresses on the growth and auxin homeostasis of Arabidopsis seedlings. BioMetals 28, 123–132. 10.1007/s10534-014-9808-6 25416404

[B125] Wu D. Shen H. Yokawa K. Baluška F. (2014). Alleviation of aluminium-induced cell rigidity by overexpression of OsPIN2 in rice roots. J. Exp. Bot. 65, 5305–5315. 10.1093/jxb/eru292 25053643 PMC4157713

[B126] Xian J. Wang Y. Niu K. Ma H. Ma X. (2020). Transcriptional regulation and expression network responding to cadmium stress in a Cd-tolerant perennial grass Poa Pratensis. Chemosphere 250, 126158. 10.1016/j.chemosphere.2020.126158 32092564

[B127] Xu N. Chu Y. Chen H. Li X. Wu Q. Jin L. (2018). Rice transcription factor OsMADS25 modulates root growth and confers salinity tolerance via the ABA–mediated regulatory pathway and ROS scavenging. PLoS Genet. 14 (10), e1007662. 10.1371/journal.pgen.1007662 30303953 PMC6197697

[B128] Yan J. Zhao C. Zhou J. Yang Y. Wang P. Zhu X. (2016). The miR165/166 mediated regulatory module plays critical roles in ABA homeostasis and response in *Arabidopsis thaliana* . PLoS Genet. 12, e1006416. 10.1371/journal.pgen.1006416 27812104 PMC5094776

[B129] Yang J. He H. He Y. Zheng Q. Li Q. Feng X. (2021). TMK1-based auxin signaling regulates abscisic acid responses via phosphorylating ABI1/2 in Arabidopsis. Proc. Natl. Acad. Sci. U. S. A. 118, e2102544118. 10.1073/pnas.2102544118 34099554 PMC8214701

[B130] Yang T. Wang Y. Teotia S. Wang Z. Shi C. Sun H. (2019). The interaction between miR160 and miR165/166 in the control of leaf development and drought tolerance in Arabidopsis. Sci. Rep. 9, 2832. 10.1038/s41598-019-39397-7 30808969 PMC6391385

[B131] Yang Y. Guo Y. (2018). Unraveling salt stress signaling in plants. J. Integr. Plant Biol. 60, 796–804. 10.1111/jipb.12689 29905393

[B132] Yoon E. K. Yang J. H. Lim J. Kim S. H. Kim S. K. Lee W. S. (2010). Auxin regulation of the microRNA390-dependent transacting small interfering RNA pathway in Arabidopsis lateral root development. Nucleic Acids Res. 38, 1382–1391. 10.1093/nar/gkp1128 19969544 PMC2831332

[B133] Yu C. Sun C. Shen C. Wang S. Liu F. Liu Y. (2015). The auxin transporter, OsAUX1, is involved in primary root and root hair elongation and in Cd stress responses in rice (Oryza sativa L). Plant J. 83, 818–830. 10.1111/tpj.12929 26140668

[B134] Yu Y. Tang W. Lin W. Li W. Zhou X. Li Y. (2023). ABLs and TMKs are co-receptors for extracellular auxin. Cell 186, 5457–5471.e17. 10.1016/j.cell.2023.10.017 37979582 PMC10827329

[B135] Yuan S. Zhao J. Li Z. Hu Q. Yuan N. Zhou M. (2019). MicroRNA396-mediated alteration in plant development and salinity stress response in creeping bentgrass. Hortic. Res. 6, 48. 10.1038/s41438-019-0130-x 31069081 PMC6491569

[B136] Zhan Y. Zhang C. Zheng Q. Huang Z. Yu C. (2017). Cadmium stress inhibits the growth of primary roots by interfering auxin homeostasis in Sorghum bicolor seedlings. J. Plant Biol. 60, 593–603. 10.1007/s12374-017-0024-0

[B137] Zhang A. Yang X. Lu J. Song F. Sun J. Wang C. (2021). OsIAA20, an Aux/IAA protein, mediates abiotic stress tolerance in rice through an ABA pathway. Plant Sci. 308, 110903. 10.1016/j.plantsci.2021.110903 34034863

[B138] Zhang H. Zhu J. Gong Z. Zhu J. K. (2022). Abiotic stress responses in plants. Nat. Rev. Genet. 23, 104–119. 10.1038/s41576-021-00413-0 34561623

[B139] Zhang N. Yu H. Yu H. Cai Y. Huang L. Xu C. (2018a). A core regulatory pathway controlling rice tiller angle mediated by the LAZY1-dependent asymmetric distribution of auxin. Plant Cell 30, 1461–1475. 10.1105/tpc.18.00063 29915152 PMC6096585

[B140] Zhang P. Sun L. Qin J. Wan J. Wang R. Li S. (2018b). cGMP is involved in Zn tolerance through the modulation of auxin redistribution in root tips. Environ. Exp. Bot. 147, 22–30. 10.1016/j.envexpbot.2017.10.025

[B141] Zhao J. Wang W. Zhou H. Wang R. Zhang P. Wang H. (2017). Manganese toxicity inhibited root growth by disrupting auxin biosynthesis and transport in Arabidopsis. Front. Plant Sci. 8, 272. 10.3389/fpls.2017.00272 28316607 PMC5334637

[B142] Zhao Y. (2010). Auxin biosynthesis and its role in plant development. Annu. Rev. plant Biol. 61, 49–64. 10.1146/annurev-arplant-042809-112308 20192736 PMC3070418

[B143] Zhao Y. (2018). Essential roles of local auxin biosynthesis in plant development and in adaptation to environmental changes. Annu. Rev. Plant Biol. 69, 417–435. 10.1146/annurev-arplant-042817-040226 29489397

[B144] Zhou Q. Y. Tian A. G. Zou H. F. Xie Z. M. Lei G. Huang J. (2008). Soybean WRKY-type transcription factor genes, GmWRKY13, GmWRKY21, and GmWRKY54, confer differential tolerance to abiotic stresses in transgenic Arabidopsis plants. Plant Biotechnol. J. 6, 486–503. 10.1111/j.1467-7652.2008.00336.x 18384508

[B145] Zhou R. Yu X. Ottosen C. O. Zhang T. Wu Z. Zhao T. (2020). Unique miRNAs and their targets in tomato leaf responding to combined drought and heat stress. BMC Plant Biol. 20, 107. 10.1186/s12870-020-2313-x 32143575 PMC7060562

